# Quantum Metal‐Organic Frameworks

**DOI:** 10.1002/smsc.202400161

**Published:** 2024-08-06

**Authors:** Zhehao Huang, Richard Matthias Geilhufe

**Affiliations:** ^1^ Center for Electron Microscopy School of Emergent Soft Matter South China University of Technology Guangzhou 510006 China; ^2^ Department of Materials and Environmental Chemistry Stockholm University 106 91 Stockholm Sweden; ^3^ Department of Physics Chalmers University of Technology 412 96 Göteborg Sweden

**Keywords:** dynamics, materials science, porous materials: metal-organic framework, quantum materials: superconductors, topological materials

## Abstract

Quantum materials and metal‐organic framework (MOFs) materials describe two attractive research areas in physics and chemistry. Yet, with very few exceptions, these fields have been developed with little overlap. This review aims to summarize these efforts and outline the huge potential of considering MOFs as quantum materials, called quantum MOFs. Quantum MOFs exhibit macroscopic quantum states over wide energy and lengths scales. Examples are topological materials and superconductors, to name but a few. In contrast to conventional quantum materials, MOFs exhibit promising unconventional degrees of freedom such as buckling, interpenetration, porosity, and rotations, stimulating the design of novel quantum phases of matter.

## Graphical abstract

Quantum metal‐organic frameworks (Q‐MOFs) merge quantum materials and MOFs, showcasing their potential in physics and chemistry. This review highlights Q‐MOFs’ macroscopic quantum states, including topological materials and superconductors, and explores their unique, unconventional degrees of freedom, such as buckling, interpenetration, and porosity, driving the innovation of new quantum phases of matter.

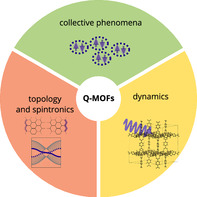

## 1 Introduction: Quantum Materials

Quantum mechanics provides a theoretical framework for describing material properties at the microscopic level. However, many materials display quantum phenomena at macroscopic scales, giving rise to the term quantum materials.^[^ ^1^ ^]^ These encompass phenomena such as superconductivity, superfluidity, topological and Dirac materials, as well as magnetism, that is, phenomena that lack a classical description. Recent extensions into the time domain include transient and temporal orders,^[^ ^2^ ^]^ time crystals,^[^ ^3^ ^]^ and Floquet systems.^[^ ^4^ ^]^

Understanding quantum materials hinges on the principle of emergence.^[^ ^5^ ^]^ Collective states formed by countless ions and electrons exhibit properties not present in individual constituents. Elementary excitations of these collective states give rise to diverse quasiparticles, often originating from a few degrees of freedom. In conventional crystalline materials, these degrees encompass the crystalline lattice, electron orbital, spin, and charge. For instance, ferromagnetism arises from the alignment of local magnetic moments, dependent on electronic spin and occupation imbalances.

The classification of states of matter, historically described by the Landau–Ginzburg–Wilson theory, faces challenges, particularly in systems where transitions don't entail symmetry breaking. Topological order exemplifies this, where phases are distinguished by topological invariants.^[^ ^6^, ^7^, ^8^, ^9^, ^10^ ^]^ Temporal orders, like transient order in nonequilibrium systems,^[^ ^11^ ^]^ further complicate traditional paradigms, prompting consideration of coherent quantum real‐time evolution.^[^ ^12^ ^]^

Besides introducing new mechanisms, considering novel materials, such as metal‐organic framework (MOFs) materials, opens new opportunities and challenges in quantum materials research. MOFs, composed of inorganic and organic components, offer tunable functionality at the molecular level through supramolecular chemistry. They find applications in catalysis, sensing, gas sorption, electronics, biomedicine, and environmental science. However, despite their potential synergy, the research fields of MOFs and quantum materials have evolved almost separately.

**Figure 1:**
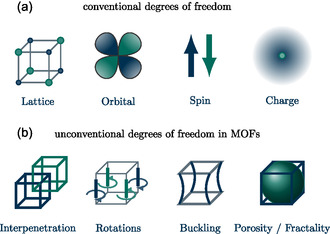
Emergence in quantum materials is controlled by internal degrees of freedom. a) Conventional quantum materials, these typically include the crystalline lattice, electron orbital, spin, and charge. b) In contrast, unconventional degrees of freedom emerge within MOFs. MOF structures can be built “within each other,” leading to interpenetrated MOFs. Furthermore, organic linkers allow for rotations or buckling. MOFs are generally porous, making them hybrids of molecules and crystals and resulting in fractional interfaces with interesting consequences on topological states of matter.

This review aims to serve as an accessible reference for researchers in MOF or quantum materials fields. It covers essential concepts in quantum materials and ongoing efforts in realizing quantum phenomena in MOFs. Here, the focus is on topological materials and superconductors. For magnetic MOFs, we refer to other excellent reviews, for example, refs. [13, 14]. Furthermore, the review explores opportunities for novel quantum phenomena based on unique properties like interpenetration, rotations, buckling, and porosity in MOFs (**Figure** **1**).

We note that the concept of quantum MOFs materials can be generalized to quantum framework materials, including, for example, covalent organic frameworks^[^ ^15^, ^16^, ^17^, ^18^ ^]^ or hydrogen‐bonded organic frameworks.^[^ ^19^, ^20^ ^]^

## 2 Frontiers in MOF Design

### 2.1 Design at a Molecular Level

MOFs or porous coordination polymers are a class of hybrid materials constructed by coordinating metal clusters/cations with organic linker molecules.^[^ ^21^, ^22^ ^]^ The wide variety of inorganic building units (IBUs) and linker molecules has resulted in an almost unlimited range of combinations for constructing MOFs. To date, more than 90 000 MOFs have been developed, offering diverse pore sizes and functionalities. Consequently, MOFs have demonstrated high potential in gas storage,^[^ ^23^, ^24^, ^25^, ^26^ ^]^ separation,^[^ ^27^, ^28^, ^29^, ^30^ ^]^ energy storage and conversion,^[^ ^31^, ^32^, ^33^, ^34^, ^35^, ^36^ ^]^ drug delivery,^[^ ^37^, ^38^ ^]^ sensing,^[^ ^39^, ^40^, ^41^, ^42^ ^]^ catalysis,^[^ ^43^, ^44^, ^45^, ^46^, ^47^ ^]^ etc. One of the most exciting features of MOFs is their design flexibility, allowing the tailoring of properties, such as pore structures, chemical functionalities, and structural flexibilities at a molecular level.^[^ ^21^, ^22^, ^48^, ^49^, ^50^, ^51^ ^]^

**Figure 2:**
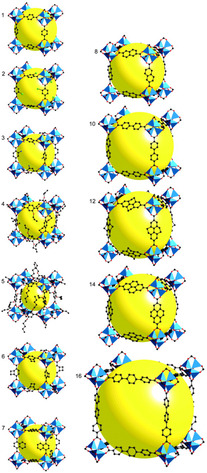
The isoreticular approach to tailor the properties of MOFs. Series of isoreticular MOFs based on MOF‐5. Reprinted with permission.^[^ ^48^ ^]^ Copyright 2002, American Association for the Advancement of Science.

Beyond the traditional approach of describing crystal structures using bond distances and angles, topological considerations take center stage in MOFs. The MOF structure can be deconstructed into geometric units, representing the molecular framework components. The shape and connectivity of these units can be simplified by a graph representation involving vertexes and edges. Based on these topologies, reticular chemistry is a common approach to assemble IBUs and linkers through strong bonds into a solid crystalline lattice, realizing the target synthesis.^[^ ^48^ ^]^ The MOF structures can be further tailored through the isoreticular principle, for example, to control pore sizes and introduce functionalities. For example, in **Figure** **2** we show a series of isoreticular MOFs based on the MOF‐5 topology.^[^ ^48^ ^]^ Here, the IBU is kept while the organic linker is substituted with molecules modifying the pore size. The Reticular Chemistry Structure Resource^[^ ^52^ ^]^ is a database of tens of thousands of networks and for indexing their corresponding MOF structures.

Synthesizing MOFs presents significant challenges compared to well‐explored quantum state systems, primarily due to difficulties in achieving high purity and low defect crystals. These challenges arise from the reversible nature of coordination bonds, necessitating strategies such as employing competing reagents to slow crystal growth and enhancing MOF purity.^[^ ^53^ ^]^ Although chemical vapor deposition (CVD) has been investigated for synthesizing high‐quality MOFs,^[^ ^54^, ^55^ ^]^ its application remains limited to a few MOF types. Recent advancements have demonstrated the precise introduction of covalently bonded structures in assembling organic nanoarchitectures through scanning probe manipulation.^[^ ^56^ ^]^ Although this approach may not be readily scalable, it offers controlled growth and high purity, making it potentially viable for quantum devices.

Exploration of MOF quantum states is in its nascent stages, with notable challenges centered on controlling buckling and mechanical instabilities. Molecular buckling can be mitigated by promoting interactions between organic molecules within the structure. For instance, in MIL‐140C, the behavior of the linker molecule 4,4’‐biphenyldicarboxylate (bpdc) varies significantly depending on its spatial arrangements; isolated bpdc molecules exhibit greater mobility compared to those engaged in *π*–*π* interactions.^[^ ^57^ ^]^ Thus, strategic design of MOF architectures to facilitate intermolecular interactions represents a promising approach for controlling buckling phenomena.

### 2.2 Unconventional Degrees of Freedom

Materials are built from a vast amount of ions and electrons. On the microscopic scale, electrons and ions carry charge, spin, and orbital degrees of freedom, which collectively contribute to the macroscopic material properties. However, the unique structure of MOFs introduces additional unconventional degrees of freedom, in contrast to well‐known inorganic quantum materials. These degrees of freedom are interpenetration, rotations, buckling, and porosity, summarized in Figure 1.

**Figure 3:**
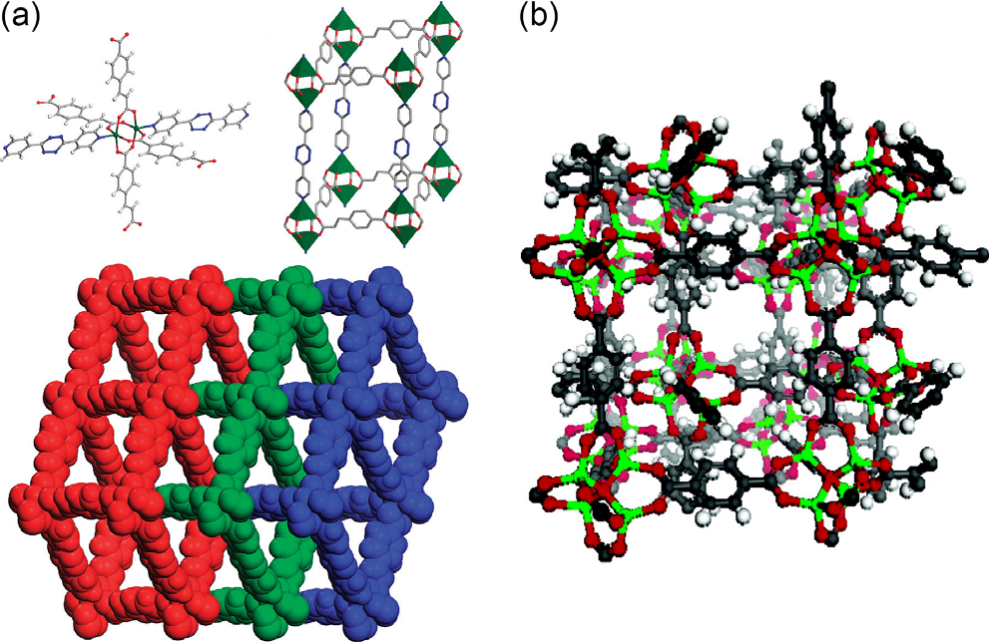
Interpenetrated MOFs. a) X‐ray crystal structure of threefold interpenetration of Zn_2_(CNC)_2_(DPT). CNC = 4‐Carboxycinnamic; DPT = 3,6‐Di‐4‐pyridyl‐1,2,4,5‐tetrazine. b) Twofold interpenetrated nets of MOF‐5 at 300 °C. (a) Reproduced with permission.^[^ ^193^ ^]^ Copyright 2008, American Chemical Society. (b) Reproduced with permission.^[^ ^194^ ^]^ Copyright 2007, American Chemical Society.

Interpenetrated frameworks describe framework structures that are not connected to each other by chemical bonds. Instead they are bound topologically, requiring the breaking of bonds in one of the structures for separation. In **Figure** **3**, we give two examples based on 1) a threefold interpenetrated structure consisting of Zn_2_(CNC)_2_(DPT) (CNC = 4‐Carboxycinnamic; DPT = 3,6‐Di‐4‐pyridyl‐1,2,4,5‐tetrazine) and 2) a twofold interpenetrated MOF‐5 structure. Interpenetrated frameworks often interact by weak forces, such as van der Waals interactions, hydrogen bonding, *π*–*π* interactions, etc., forming ordered crystal structure. The weak interaction between the frameworks allows for framework flexibility such as different spacing between the subnets or subnet sliding. The prospect of topologically entangled compounds with weak interaction opens an interesting prospect for designing novel quantum phases of matter. While interfaces between different quantum materials are investigated in great detail, interpenetration allows for the 3D coexistence of several weakly interacting quantum phases in the same volume.

Flexible MOFs, or soft porous crystals, present structural flexibility as a unique characteristic.^[^ ^49^, ^58^, ^59^, ^60^, ^61^ ^]^ Local flexibilities, such as linker rotation and swing, are common. They can be tuned by the change of spatial alignment of a linker around a rotational axis. ZIF‐8 is the prototype MOF that led to the discovery of such flexibilities, where the rotational linker movement results in the expansion of pores. As a result, ZIF‐8 is capable of capturing molecules larger than the crystallographically determined pore size.^[^ ^62^ ^]^ Interestingly, molecular rotations emerge collectively throughout the MOF structure, with distinct frequencies, for example, symmetric and antisymmetric modes, typically in the low THz regime.^[^ ^63^ ^]^

MOFs also display mechanical instabilities such as molecular buckling, emerging when the axial load on a flexible organic linker reaches a sufficient strength. Such a buckling can emerge intrinsically by the mass and composition of the MOF, or it can be induced by applying strain. Similar to vibronic or phononic excitations in molecules and crystals, collective buckling modes can be excited, which in turn interact with electronic degrees of freedom.^[^ ^64^ ^]^ Furthermore, the buckling of molecules can undergo a quantum transition at low temperatures, where a tunneling between degenerate buckling states is allowed.

The porosity of MOF structures is a key feature for chemical applications. In the context of quantum materials, it allows for embedding guest molecules mediating desired properties. The open structure challenges the well‐defined concepts of bulk and boundary, allowing for designing novel topological phases of matter and topological catalysts. Furthermore, the structural porosity allows for characteristic flexibility, such as breathing and swelling, which involves reversible crystal‐to‐crystal or crystal‐to‐amorphous structural transformations. For example, the breathing in MIL‐53 features a dramatic change in unit cell volume and pore volume between its open pore form and closed pore form, where the bond angles can change drastically upon guest molecule adsorption and desorption.^[^ ^58^ ^]^

### 2.3 Synthesis and Characterization

As most MOFs are crystalline materials, their synthesis is often guided by classic crystal growth theory. Solution crystal growth methods, such as hydrothermal, solvothermal, and ionothermal synthesis, are the predominant ways in MOF synthesis. Moreover, interfacial synthesis is one of the most widely used approaches for 2D MOFs. In this method, the reactions between metals and organic linkers occur at interfaces (liquid/air, liquid/liquid, and liquid/solid).^[^ ^65^, ^66^ ^]^ Due to the confined 2D interfacial region, the resulting MOFs often exhibit a nanosheet morphology.

In addition to bottom‐up methods, postsynthetic approaches are prominent. For example, solvent‐assisted linker exchange is a method for the postsynthesis of MOFs.^[^ ^67^, ^68^ ^]^ It involves heterogeneous reactions of parent MOF crystals with a concentrated solution of linkers. The linkers from the solution are then incorporated into a framework by substituting linkers from the parent crystal. As a result, the synthesized MOFs possess the same topology as the parent crystals.

Apart from synthesis approaches in solutions, MOFs can also be prepared by solid‐solid synthesis methods. Mechanochemical approaches provide solvent‐free pathways for preparing MOFs, which also offer easier scalability.^[^ ^69^ ^]^ By avoiding the use of organic solvents, it improves the environmental aspect of MOF synthesis and provides a greener approach compared to conventional MOF synthesis.

MOF crystal structures are routinely resolved by single‐crystal X‐ray diffraction. For MOFs with small crystal sizes, that is, in the range of nanometers, powder X‐ray diffraction and the recently developed 3D electron diffraction^[^ ^70^ ^]^ are complementary approaches.

## 3 Topology and Spintronics

### 3.1 A Brief Overview

Despite the framework topology in MOFs, electronic states in the crystalline lattice can be classified according to their topology. Here, a nontrivial topology of electrons is accompanied by striking transport properties. The first discovered topological quantum state was the quantum Hall effect, found by Klaus von Klitzing.^[^ ^71^ ^]^ von Klitzing identified quantized Hall plateaus in Hall voltage measurements of a 2D electron gas. Quickly, this remarkable discovery led to early theoretical work, unraveling the topological origin of the quantum Hall effect.^[^ ^72^, ^73^ ^]^

The theoretical breakthrough came with the work by Thouless, Kohmoto, Nightingale, and den Nijs in 1982.^[^ ^74^ ^]^ Extending linear response theory, they showed that the Hall conductance is quantized and independent of the actual shape of the band structure. Instead, the Hall conductance *σ*_*ij*_ relies on a topological invariant, the Chern number *C*_α_ of occupied states *α*,^[^ ^75^ ^]^ expressed by

(1) $$\sigma_{xy}=-\frac{e^{2}}{2\pi \hbar }\sum_{\alpha \;\text{occ.}}C_{\alpha }$$

Here, *e* is the electronic charge and *ℏ* the Planck constant. The Chern number is expressed in terms of the Bloch wave functions $\Psi_{\alpha \vec{k}}(\vec{r})=e^{i\vec{k}\cdot \vec{r}}u_{\alpha \vec{k}}(\vec{r})$

(2) $$C_{\alpha }=\frac{i}{2\pi }\int d^{2}k\;\frac{\partial u_{\alpha \vec{k}}^{*}}{\partial k_{x}}\frac{\partial u_{\alpha \vec{k}}}{\partial k_{y}}-\frac{\partial u_{\alpha \vec{k}}^{*}}{\partial k_{y}}\frac{\partial u_{\alpha \vec{k}}}{\partial k_{x}}$$

**Figure 4:**
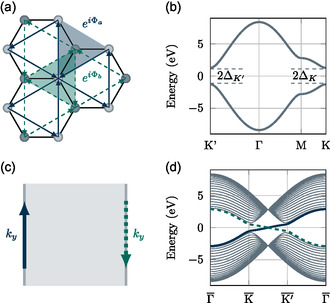
The Haldane model. a) Next‐nearest neighbor hopping terms and nonzero flux leading to time‐reversal symmetry breaking. b) Bulk band structure showing two topological gaps at *K* and *K*′. c) Illustration of the two edges occurring for a finite 2D stripe. The arrows indicate the momenta of the two edge currents expected in the Haldane model. d) Band structure calculation for a finite stripe, showing the crossing of the two edge bands in the topological state$sign(\Delta_{K})=-sign(\Delta_{-K})$.

Shortly after, Haldane^[^ ^76^ ^]^ showed theoretically the existence of an anomalous quantum Hall effect (**Figure** **4**). The model is based on a honeycomb lattice as shown in Figure 4a with broken time‐reversal invariance (note that a magnetic field also breaks time‐reversal symmetry). The electronic band structure, shown in Figure 4b, can be approximated by an effective Hamiltonian for the *K* and *K*′ points in the Brillouin zone.

(3) $$H(\vec{K}+\vec{k})\approx \hbar v_{D}\left(\sigma_{x}k_{x}+\sigma_{y}k_{y}\right)+\Delta_{K}\sigma_{z}$$

(4) H(K→'+k→)≈ℏvD(σxkx−σyky)+ΔK′σz

Here, *σ*_*i*_ (*i* = *x*, *y*, *z*) are the three Pauli matrices, acting in sublattice space, *v*_D_ is the so‐called Dirac velocity, a model‐specific parameter, and $\Delta_{\alpha }$ ($\alpha =K,K^{\prime }$) the gap. Note that by time‐reversal symmetry, *K* is mapped to *K*′. Hence, if $\Delta_{K}=\Delta_{K^{\prime }}$ the model is time‐reversal invariant. Equation (3) and (4) have the form of a Dirac equation, for which the Chern number of the lower branch can be evaluated as follows

(5) $$C=-\frac{1}{2}sign(v_{D})sign(\Delta_{\alpha })$$

Hence, adding the contribution of both *K* and *K*′ to the Hall conductance, one obtains $\sigma_{xy}=\frac{e^{2}}{2\pi \hbar }\nu$, with

(6) $$\nu =\frac{1}{2}\left(sign(\Delta_{K^{\prime }})-sign(\Delta_{K})\right)$$

From Equation (6), it becomes apparent that the Hall conductance vanishes in the time‐reversal symmetric case, that is, $\Delta_{K^{\prime }}=\Delta_{K}$. Interestingly, the emergence of the Hall conductance is also reflected in the level crossing of edge states, as shown in Figure 4d. This phenomenon is referred to as bulk‐boundary correspondence, that is, the topological features are determined in the bulk, but observed on the boundary.

While Haldane's model was insightful at the time, its realization seemed far. It almost took another 20 years until the first synthesis of graphene^[^ ^77^ ^]^ and the era of 2D materials. Only then, topological systems were found to exist in the presence of time‐reversal symmetry. For example, Kane and Mele predicted that graphene, the prototypical Dirac material, has to exhibit a tiny gap opened by spin‐orbit interaction.^[^ ^78^ ^]^ Compliant with all lattice symmetries this gap takes the form

(7) $$H_{\text{SO}}=\Delta \;\sigma_{z}\tau_{z}s_{z}$$

with the Pauli matrix $\tau_{z}$ acting in “valley” space (i.e., *K* and *K*′) and the Pauli matrix *s*_*z*_ acting in spin space. Since the signs of the gaps at *K* and *K*′ are opposite for the opposite spin channels, a net spin current emerges as soon as an electric field is applied, ${\vec{J}}_{s}=\frac{\hbar }{2e}({\vec{J}}_{↑}-{\vec{J}}_{↓})$. This current is characterized by a quantized spin‐Hall conductivity

(8) $$\sigma_{xy}^{s}=\frac{e}{2\pi }$$

In contrast, the charge Hall effect is zero. This finding has been an important cornerstone in the development of spintronics, where technology is built based on the electron spin.

Independently of Kane and Mele, the quantum spin‐Hall effect was found by Bernevig, Hughes, and Zhang (BHZ), proposing a 2D topological model realized in HgTe quantum wells.^[^ ^79^ ^]^ Again, BHZ included spin to recover time‐reversal symmetry. For a Chern insulator describing the Hamiltonian $H(\vec{k})$, the following time‐reversal invariant Hamiltonian can be formulated.

(9) $$H_{\text{BHZ}}=\left(\begin{array}{cc} H(\vec{k}) & 0\\ 0 & H^{*}(-\vec{k}) \end{array}\right)$$

Note that in the present example, time‐reversal symmetry acts as a combination of $\vec{k}\to -\vec{k}$, and the operator $is_{y}K$, with *s*_*y*_ acting in spin space and *K* being the complex conjugation. As in the Kane–Mele model, the Chern numbers concerning the blocks $H(\vec{k})$ and $H^{*}(-\vec{k})$ are of opposite sign. As a result, the quantized Hall conductance for the charge vanishes. Instead, its difference gives a nonzero quantized spin‐Hall conductance.

The extension of the above approaches to 3D followed soon after.^[^ ^80^, ^81^, ^82^ ^]^ The first experimental realization of a 3D topological insulator was found in Bi_1−*x*_Sb_*x*_ using angle‐resolved photoemission spectroscopy.^[^ ^83^ ^]^ Subsequently, the topological insulator phase was also seen in the stochiometric compounds Bi_2_Se_3_, Bi_2_Te_3_, and Sb_2_Se_3_.^[^ ^84^, ^85^, ^86^ ^]^ A variant of the topological insulator, based on the so‐called mirror Chern number, was found in Pb_1−*x*_Sn_*x*_Se.^[^ ^87^ ^]^ For more specialized reviews about topological insulators, we refer to refs. [88, 89].

### 3.2 2D Topological MOFs

While the symmetry aspects of topological materials have been intensively discussed by various communities, both in 2D and 3D, the realization of topological electronic states in MOFs is dominated by 2D structures.

Recently, 2D materials have garnered considerable interest, beginning with graphene and expanding to include a diverse array of materials such as transition metal dichalcogenides, group‐VIB dichalcogenides, phosphorene, group‐IV monochalcogenides, gallium and indium monochalcogenides, hexagonal boron nitride, oxide layers, and 2D magnets like FePS_3_, CrI_3_, Cr_2_Ge_2_Te_6_,^[^ ^90^, ^91^, ^92^, ^93^, ^94^, ^95^, ^96^, ^97^ ^]^ and 2D MOFs.^[^ ^98^ ^]^ Consequently, experimental techniques have been developed to fabricate van der Waals bonded heterostructures, twisted configurations, and to engineer strain.^[^ ^90^, ^91^, ^92^, ^93^, ^99^ ^]^ Here, local probing techniques such as scanning tunneling microscopy (STM) have been important for characterizing structure and electronic properties.^[^ ^100^, ^101^, ^102^, ^103^, ^104^ ^]^ This development has also contributed to identifying materials potentially hosting a quantum anomalous Hall state, such as magnetically doped topological insulators, intrinsically magnetic topological insulators, or Moire materials.^[^ ^105^ ^]^

Translating concepts known from 2D materials to 2D MOFs, the Liu group pioneered the field of topological MOFs in a series of theoretical papers in 2013, showcasing the first MOF topological insulator,^[^ ^106^, ^107^ ^]^ the quantum anomalous Hall effect in MOFs,^[^ ^108^ ^]^ and topological flat bands in MOFs.^[^ ^109^ ^]^ 2D MOFs show the prospect of clean and controllable synthesis as well as embedding into devices.

**Figure 5:**
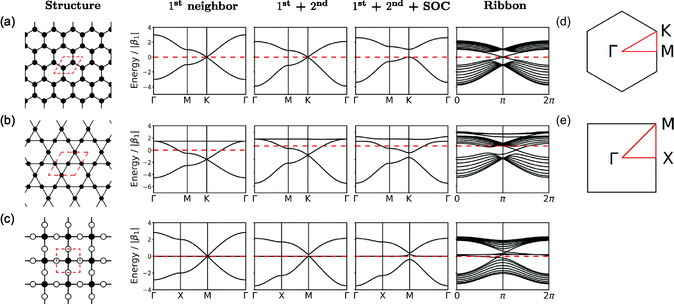
Generic electronic structure of a) hexagonal lattice, b) Kagome lattice, and c) Lieb lattice. d,e) Brillouin zones for (a,b) as well as (c), respectively. Reproduced with permission.^[^ ^110^ ^]^ Copyright 2007, Royal Society of Chemistry.

To show the intricate connection between structural topology and electronic topology in 2D MOFs and polymers, Springer et al.^[^ ^110^ ^]^ discussed periodic tesselations, that is, tilings of the 2D plane using geometric shapes, such as squares, triangles, hexagons. In doing so, they compare generic tight‐binding band structures for the nearest‐neighbor approximation, the second‐nearest neighbor approximation, the effect of spin orbit interaction, and the band structure of a finite ribbon for a total amount of 101 2D networks. A closely related work was published by Jiang et al.^[^ ^111^ ^]^ focusing on the topological properties of the honeycomb, Kagome, and Lieb lattices, as well as extensions thereof. In fact, it is in particular these three lattices which have been in the focus of theoretical and experimental efforts summarized subsequently (compare **Figure** **5**). We also mention Fan et al. as a great reference summarizing tight‐binding models for these lattices.^[^ ^112^ ^]^

The honeycomb lattice contains two sites in the elementary unit cell (Figure 5a). If one orbital degree of freedom is considered per site, the simplest model gives rise to two bands. Without spin‐orbit coupling, these bands cross linearly (Dirac crossings) at the *K* and *K*′ points in the Brillouin zone (Figure 5d). With spin‐orbit coupling, a topological gap can be opened at *K* and *K*′ prime, leading to a linear crossing of bands of edge states in a ribbon (similar to the Kane–Mele model before).

A realization of a MOF with honeycomb lattice was theoretically predicted by Wang et al.^[^ ^108^ ^]^ considering triphenyl transition‐metal networks. They assumed a framework composed of [Mn(C_6_H_5_)_3_] molecules (TMn). The ground state is ferromagnetic, with a magnetization of 2 *μ*_B_ per Mn atom. The finite magnetization in combination with local atomic spin‐orbit interaction of the Mn atoms in the framework induces a quantum anomalous Hall effect, related to the Haldane model. Interestingly, the topological gap lies right at the Fermi level, with a computational value of 9.5 meV.

Liu et al.^[^ ^109^ ^]^ predicted the properties of a 2D indium‐phenylene organometallic framework. The electronic structure of the framework around the Fermi level is dominated by in‐plane vectorial orbitals (*p*_*x*_, *p*_*y*_ belonging to C and In). The interplay of these orbitals with spin‐orbit interaction, a finite magnetization, and *p*‐doping induces a nearly flat band with a finite Chern number close to the Fermi level.

Hsu et al.^[^ ^113^ ^]^ performed a theoretical study, predicting the stability and electronic structures of organometallic frameworks consisting of metal atoms (elements from groups IIIA, IVA, VA, VIA, IB, and Pt) and dicyanobenzenes (DCBs). This work revealed topological phases with a nontrivial *Z*_2_ invariant for the metal ions Au, Ag, Cu, In, Tl, Sn, Pb, Sb, and Bi. Specific attention is given to Au‐DCB exhibiting a quantum spin‐Hall gap of 14.3 meV and Bi‐DCB, exhibiting a tiny quantum anomalous Hall gap of 1 meV.

The Kagome lattice contains three sites in the elementary unit cell (Figure 5b) giving rise to a minimal model with three bands. One band is flat, while the other two bands are dispersive, showing a linear crossing at the *K* and *K*′ points in the Brillouin zone (Figure 5d). Spin‐orbit coupling can lift this degeneracy to open a topological gap with linearly crossing edge modes for a finite ribbon.

Wang et al.^[^ ^107^ ^]^ analyzed the π‐conjugated nickel‐bis‐dithiolene, Ni_3_C_12_S_12_ predicting its band structure to exhibit topological gaps at the *K* and *K*′ points ($\Delta_{1}=13.6\text{meV}$) as well as between a Dirac band and the flat band ($\Delta_{2}=5.8\text{meV}$). The finite magnetization of the sample breaks the time‐reversal symmetry and leads to a quantum anomalous Hall state. Interestingly, Ni_3_C_12_S_12_ was synthesized before.^[^ ^66^ ^]^

A closely related work was published by Zhao et al.^[^ ^114^ ^]^ predicting the electronic structure of Ni_3_(C_18_H_12_N_6_)_2_, inspired by the synthesis of the corresponding layered structure.^[^ ^115^ ^]^ Here, however, the quantum anomalous Hall state lies above the Fermi level. Zhao et al.^[^ ^114^ ^]^ show that the quantum anomalous Hall gap at *K* and *K*′ could be accessed by doping with 2 electrons per elementary cell.

Gao et al.^[^ ^116^ ^]^ reported about the successful synthesis of the 2D MOF Ni_3_(HITP)_2_, grown on a Au(111) surface. The accompanying theoretical band structure calculations based on density functional theory claim the stability of a topological gap of ≈10 meV, which lies ≈0.5 eV above the Fermi energy.

**Figure 6:**
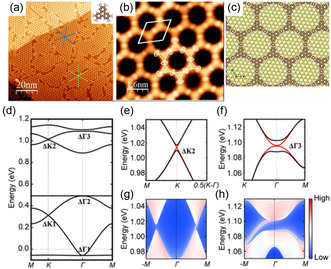
Synthesis and characterization of the 2D MOF Fe_3_(HITP)_2_, as well as electronic structure prediction revealing several topological gaps in the Brillouin zone. a,b) Scanning tunneling microscopy images from the synthesized structure. c) The theoretical framework structure. d–f) The computed electronic with (black) and without (red) spin‐orbit interaction. g,h) The emergence of edge states for a finite ribbon. Reproduced with permission.^[^ ^117^ ^]^ Copyright 2020, American Chemical Society.

In a follow‐up, Gao et al.^[^ ^117^ ^]^ reported on the synthesis and theoretical analysis of the 2D MOF Fe_3_(HITP)_2_ (see **Figure** **6**). The theoretical part of their paper predicts several topological gaps with anomalous quantum Hall states at the edge. However, all of these gaps are located ≈1 eV above the Fermi level. Hence, these states could not be accessed experimentally.

The Lieb lattice contains three sites in the elementary unit cell (Figure 5c), resulting in three bands for a minimal model. One band is flat, while the two bands are dispersive, with a linear crossing at the *M* point in the Brillouin zone (Figure 5e). Spin‐orbit coupling can lift this degeneracy to open a topological gap with edge states connecting the three bands for a finite ribbon.

Jiang et al.^[^ ^16^ ^]^ theoretically predicted the electronic structure of a phthalocyanine‐based MOF (MPc‐MOF) using density functional theory. They show that a trivial Lieb band structure with 1/3 filling can be tuned into a topological phase by applying strain or involving chemical substitution. In particular, they reveal a transition into a Chern insulator at ≈4% strain and a time‐reversal broken quantum spin Hall state^[^ ^118^ ^]^ at ≈5.5%.

### 3.3 Topology versus Strong Interactions

Various 2D framework structures are predicted or synthesized on lattices realizing topological gaps. These gaps are typically opened by spin‐orbit interaction, while linear band crossings are observed when spin‐orbit interaction is turned off (theoretically). For the case where such crossings coincide with the Fermi energy, the low‐energy physics is dominated by electrons with a relativistic energy–momentum relation $E_{\pm }(\vec{k})=\pm \hbar v_{D}\left|\vec{k}\right|$, with *ℏ* the Planck constant and *v*_D_ the Dirac velocity, being the slope of the energy–momentum relation. In physical terms, $E_{\pm }$ corresponds to the kinetic energy of electronic excitations in the frameworks.

A competing mechanism for opening a trivial gap is given by spontaneous symmetry breaking, which occurs for strong interactions between electronic excitations. The strength of these interactions is described by the effective fine‐structure constant *α*, being the ratio of Coulomb interaction and kinetic energy.

(10) $$\alpha =\frac{e^{2}}{\varepsilon \hbar v_{D}}$$

Here, *e* is the elementary charge and *ε* the dielectric constant. In quantum electrodynamics, where *v*_D_ is given by the speed of light, the fine structure constant is small, $\alpha \approx \frac{1}{137}$. As a result, interactions can be treated perturbatively. In contrast, in materials where *v*_D_ is small compared to the speed of light, interaction effects become considerable. This has been discussed intensively, for example, for graphene^[^ ^119^, ^120^, ^121^, ^122^, ^123^, ^124^, ^125^ ^]^ and other Dirac materials.^[^ ^126^, ^127^, ^128^, ^129^, ^130^, ^131^, ^132^, ^133^ ^]^ Furthermore, spontaneous symmetry breaking is a well‐studied concept in elementary particle theory to describe mechanisms for fermionic mass generation.^[^ ^134^, ^135^ ^]^ We note that the prototypical 2D Dirac material graphene has an effective fine structure constant in the order of $\alpha^{\text{graphene}}\approx 1$.^[^ ^119^ ^]^ In **Table** **1**, we deduced the Dirac velocities for various 2D MOFs mentioned above, compared to the Dirac velocity of graphene. As the Dirac velocities in MOFs tend to be smaller by at least a factor of 2, interaction effects in 2D MOFs should become dominant. As a result, the band structure is altered by establishing a trivial gap due to spontaneous symmetry breaking.

**Table 1:** Dirac velocities for several MOFs compared against the Fermi velocity of graphene $v_{D}^{\text{graphene}}\approx 1\times 10^{6}{\text{ms}}^{-1}$.

Material	References	$v_{D}/v_{D}^{\text{graphene}}$
TMn	[108]	0.18
Bi‐DCB	[113]	0.53
Au‐DCB	[113]	0.39
Ni_3_C_12_S_12_	[107]	0.42
Ni_3_(C_18_H_12_N_6_)_2_	[114]	0.58
Ni_3_(HITP)_2_	[116]	0.58
Fe_3_(HITP)_2_	[117]	0.21
MPc‐MOF	[111]	4.80

One of the simplest models to describe the spontaneous symmetry breaking is the Gross–Neveu model.^[^ ^119^, ^122^, ^123^, ^124^, ^125^ ^]^ Here, in the low‐energy limit, the electronic excitations can be described the following effective action

(11) $$S[\bar{\Psi },\Psi ]=\int d^{d}x\;\bar{\Psi }(i\partial )\Psi +\frac{1}{2}g^{2}{\left(\bar{\Psi }\Psi \right)}^{2}$$

where *Ψ* is a four‐component spinor, $\partial =\gamma^{\mu }\partial_{\mu }$, and *g* ^2^ is a general coupling constant. In the mean‐field approximation, one can split the four‐fermion term ${\left(\bar{\Psi }\Psi \right)}^{2}$ and introduce an auxiliary field *σ*, as follows.

(12) $$S\left[\bar{\Psi },\Psi ,\sigma \right]=\int d^{d}x\;\bar{\Psi }\left(i\partial -\sigma \right)\Psi -\frac{1}{2g^{2}}\sigma^{2}$$

By integrating out the fermionic components, one obtains the action S[σ], only dependent on the auxiliary field *σ*. ApMinimizing this action with respect to *σ*, $\frac{\partial S[\sigma ]}{\partial \sigma }=0$, gives rise to a self‐consistent equation for *σ*. In 2 + 1 dimensions this equation is given by

(13) $$\sigma \approx \frac{g^{2}}{2\pi^{2}V}\int_{0}^{\infty }dp\;\frac{\sigma p^{2}}{p^{2}+\sigma^{2}}$$

For a slowly varying field $\sigma (\vec{r},t)\approx \sigma$, this integral is divergent, but can be solved up to an upper momentum cutoff Λ.

(14) $$\sigma \approx \frac{g^{2}\sigma }{2\pi^{2}V}\left[\Lambda -\frac{|\sigma |\pi }{2}\right]$$

Here, *V* denotes the unit cell volume. Solving (14) for |σ| gives

(15) $$|\sigma |=\frac{2}{\pi }\left[\Lambda -\frac{2\pi^{2}V}{g^{2}}\right]$$

**Figure 7:**
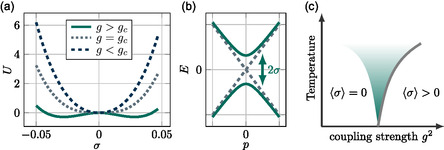
Spontaneous symmetry breaking in the mean‐field Gross‐Neveu model. a) Above a critical coupling, the action is minimized for ⟨σ⟩≠0. b) A finite value of ⟨*σ*⟩ gives rise to the opening of a gap for linearly crossing bands. c) Phase diagram with a quantum phase transition between a linearly crossing band structure and spontaneous symmetry breaking above a critical value for the coupling strength *g*_c_.

Since |σ| is a positive number, we define a critical coupling strength $g_{c}^{2}$ given by $\Lambda g_{c}^{2}=2\pi^{2}V$. For a weak coupling, $g<g_{c}$ only the trivial solution *σ* = 0 is allowed and the fermionic system remains gapless with an energy momentum relation given by $E=\pm \left|\vec{p}\right|$. In contrast, for $g>g_{c}$, the action is minimized by accumulating a finite mass σ≠0, as shown in **Figure** **7**a. As a result, a nonzero value of *σ* opens a gap in the linearly crossing band structure, with an energy momentum relation of $E=\pm \sqrt{{\vec{p}}^{2}+\sigma^{2}}$ (see Figure 7b). The phase diagram showing the quantum phase transition from a material with linearly crossing bands to a material with spontaneous symmetry breaking above a critical coupling strength is shown in Figure 7c.

### 3.4 Topology and Catalysis

By the very nature of their porous structures, MOFs have attracted significant attention as catalysts. The sparse frameworks allow the diffusion of reactants into the MOF structures, leading to a significantly increased reactive area, compared to conventional catalysts, where catalytic reactions take place on the surface. As a result, several prototype studies developed dedicated MOF catalysts for reactions of high interest,^[^ ^27^, ^136^, ^137^, ^138^ ^]^ for example, the hydrogen evolution reaction, oxygen evolution reaction, organic synthesis. For example, for the hydrogen evolution reaction, an electrolysis setup is used, where the catalytic MOF is coated or precipitated (during the reaction) on the electrode. The reaction requires metallic MOFs, which can be obtained by,^[^ ^139^ ^]^ for example, continuous bonds between inorganic building blocks and linkers, electron delocalization between inorganic building blocks and linkers, *π*–*π* stacking of organic moieties, or, guest entities. It was shown by several groups that various MOFs achieve similar catalytic performance in the hydrogen evolution reaction, compared with standard catalysts (mostly Ni), both, for 2D and 3D framework structures.^[^ ^140^, ^141^, ^142^, ^143^ ^]^ Independently of MOFs, topological materials have recently entered the field of catalysis. The topological surface states, paired with giant electron mobilities, make topological materials ideal candidates for catalysis reactions.^[^ ^144^, ^145^ ^]^ For example, Hosono et al.^[^ ^146^ ^]^ showed that the 3D topological insulator Bi_2_Se_3_ showed excellent catalytic activity for oxidative carbonylation of amines to synthesize urea derivatives. Katz et al.^[^ ^147^ ^]^ considered the hydrogen evolution reaction and concluded that due to the electronic topology, the material MoTe_2_ maintained a high carrier mobility and catalytic activity even after application. This phenomenon can be traced back to the bulk‐boundary correspondence, where the surface states in topological materials are a result of the topology of the bulk and therefore sustain (to some extent) even if the surface becomes imperfect.

**Figure 8:**
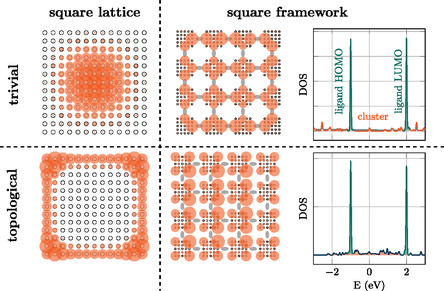
Model for a MOF containing trivial or topological inorganic building blocks, in the framework of the BHZ model. The organic linkers are modeled by a large HOMO–LUMO‐gapped two‐state system. In the trivial phase, the framework localizes electronic states (at the Fermi level) at the sites, responsible for the bonding with the organic linkers. In contrast, in the topological phase, electronic states localize at the corners of the inorganic building blocks, pointing toward the pores. This behavior might be particularly relevant for catalysis applications, where an overlap of electronic states in the MOF (catalyst) with the electronic states of the reactants (diffusing into the pores) is desired.

Up to this point, we discussed electronic topology as a global macroscopic property of a material. As the topological properties of a material also persist down to a few unit cells, we argue that MOFs also allow for linking topological IBUs with trivial molecules. We illustrate this idea in **Figure** **8**. We use the BHZ model of equation 9 to generate a generic 2D square lattice. For a lattice consisting of 15 × 15 atoms, the localization of the electronic wave function on the surface (in the bulk) within the topological phase (trivial phase) can clearly be seen for a state at the Fermi level (Figure 8 left). In the second step, we reduce the size to a 5 × 5 lattice coordinated by trivial molecules, modeled by $\Delta E\tau_{z}$, with Δ*E* being the HOMO–LUMO gap of the linker molecule. Using this model, we determine for a trivial inorganic cluster that electrons around the Fermi level localize at the bonds to the organic linker molecules. In contrast, electrons of topological inorganic building blocks tend to localize in the direction of the pores (Figure 8 right). As this is exactly the position where a catalytic reaction takes place, we see the construction of MOFs consisting of topological inorganic building blocks as a promising path towards designing novel MOF catalysts.

## 4 Collective Phenomena

### 4.1 Superconductivity

Superconductivity is one of the most striking macroscopic phenomena. In the superconducting phase, electrons condense into a collective state with vanishing resistivity. After its discovery by Kamerlingh Onnes in 1911, it could only be described in terms of a phenomenological theory by Ginzburg and Landau in 1950^[^ ^148^ ^]^ and, in terms of a microscopic theory by Bardeen, Cooper, and Schrieffer in 1957 (BCS theory).^[^ ^149^ ^]^ In 1959, Gor'kov showed that both descriptions are equivalent.^[^ ^150^ ^]^ A cornerstone in the understanding of superconductors was the isotope effect discovered by Maxwell in 1950.^[^ ^151^ ^]^ Maxwell could show that the transition temperature of natural mercury ($T_{c}=4.156$ K) is slightly lower than the transition temperature of the lighter isotope Hg^198^ ($T_{c}=4.177$ K). This pointed BCS toward an attractive electron–electron interaction mediated by phonons, giving rise to the electron condensation below the superconducting transition temperature.

Unconventional superconductors are more complex materials, where the electron condensation is a result of the strong electron–electron correlation. This has first been observed in the heavy fermion materials, for example, CeCu_2_Si_2_,^[^ ^152^ ^]^ and later in the organic charge transfer salts^[^ ^153^ ^]^ and the high‐*T*_c_ superconductors.^[^ ^154^ ^]^ Here, the strong correlations of electronic excitations typically lead to an instability prior to the superconducting phase, for example, the transition into an insulating ferro‐ or antiferromagnet. Decreasing the temperature further, one of the widely described pairing mechanisms for the superconductivity is based on spin fluctuations.^[^ ^155^, ^156^, ^157^ ^]^

**Figure 9:**
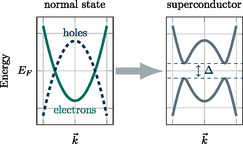
Illustration of the superconducting phase transition. Below a critical temperature, a superconducting gap Δ is established which mixes electron and hole states.

The superconducting order parameter is given by a pairing of two fermions $\Psi_{\alpha \vec{k}}$, by considering momentum conservation, ${\hat{\Psi }}_{\alpha \beta }(\vec{k})\;=\;\left\langle \Psi_{\alpha \vec{k}}\Psi_{\beta -\vec{k}}\right\rangle$. The mean‐field picture of superconductivity is shown in **Figure** **9**, for the example of a single spin‐degenerate band with (parabolic) dispersion $\varepsilon_{\vec{k}}$. At zero temperature, this band is occupied with electrons up to the Fermi level *E*_F_ and unoccupied above. In contrast, holes (i.e., unoccupied electronic states) show the inverted picture. The superconducting state is based on a mixture of electrons and holes, leading to a new type of quasi‐particle, called Bogoliubov quasiparticle. The situation is modeled using the Bogoliubov‐de‐Gennes Hamiltonian.

(16) $$H^{\text{BdG}}={\left(\begin{array}{c} {\vec{\Psi }}_{\vec{k}}^{†}\\ {\vec{\Psi }}_{-\vec{k}} \end{array}\right)}^{T}\left(\begin{array}{cc} \varepsilon_{\vec{k}} & \Delta_{\vec{k}}\\ \Delta_{\vec{k}}^{†} & -\varepsilon_{\vec{k}} \end{array}\right)\left(\begin{array}{c} {\vec{\Psi }}_{\vec{k}}\\ {\vec{\Psi }}_{-\vec{k}}^{†} \end{array}\right)$$

Here, ${\vec{\Psi }}_{\vec{k}}^{†}=(c_{↑\vec{k}}^{†},c_{↓-\vec{k}}^{†})$ is a spinor, with $c_{\sigma \vec{k}}^{†}$ being the creation operator, for creating a fermion of momentum $\vec{k}$ and spin *σ*, $\Delta_{\vec{k}}$ is called the superconducting gap function. A spin‐singlet gap corresponds to the condensation of fermions with opposite spin and is given in terms of a scalar field $\phi (\vec{k})$.

(17) $$\Delta_{\vec{k}}^{\text{singlet}}=i\sigma_{y}\phi (\vec{k})$$

In contrast, a spin‐triplet gap describes the condensation of fermions with the same spin, which is written using the pseudovector $\vec{d}(\vec{k})$.

(18) $$\Delta_{\vec{k}}^{\text{triplet}}=i\left(\vec{\sigma }\cdot \vec{d}(\vec{k})\right)\;\sigma_{y}$$

The field $\phi (\vec{k})$ and the pseudovector $\vec{d}(\vec{k})$ can be expressed in terms of spherical harmonics. This expression gives rise to the terminology of *s*‐wave, *p*‐wave, or *d*‐wave gap, etc. The classification of the superconducting gaps, based on symmetry, has been reported in great detail by various authors and summarized, for example, in the review by Sigrist and Ueda.^[^ ^158^ ^]^ For a more detailed introduction into the formalism of superconductivity, we refer to the first part of the review by Balatsky et al.^[^ ^159^ ^]^

In the normal state, where $\Delta_{\vec{k}}=0$, the Bogoliubov‐de‐Gennes Hamiltonian becomes diagonal and resembles the conventional band structure shown in Figure 9 (left). Below the superconducting transition temperature, where $\Delta_{\vec{k}}\neq 0$, the energy eigenvalues of the system change to $E_{\vec{k}}=\pm \sqrt{\varepsilon_{\vec{k}}^{2}+\frac{1}{2}Tr\Delta_{\vec{k}}\Delta_{\vec{k}}^{†}}$, as shown in Figure 9 (right). As a result, the total energy of the system is lowered by establishing a nonzero $\Delta_{\vec{k}}$, given that thermal fluctuations are weak enough to keep the system in this state.

**Figure 10:**
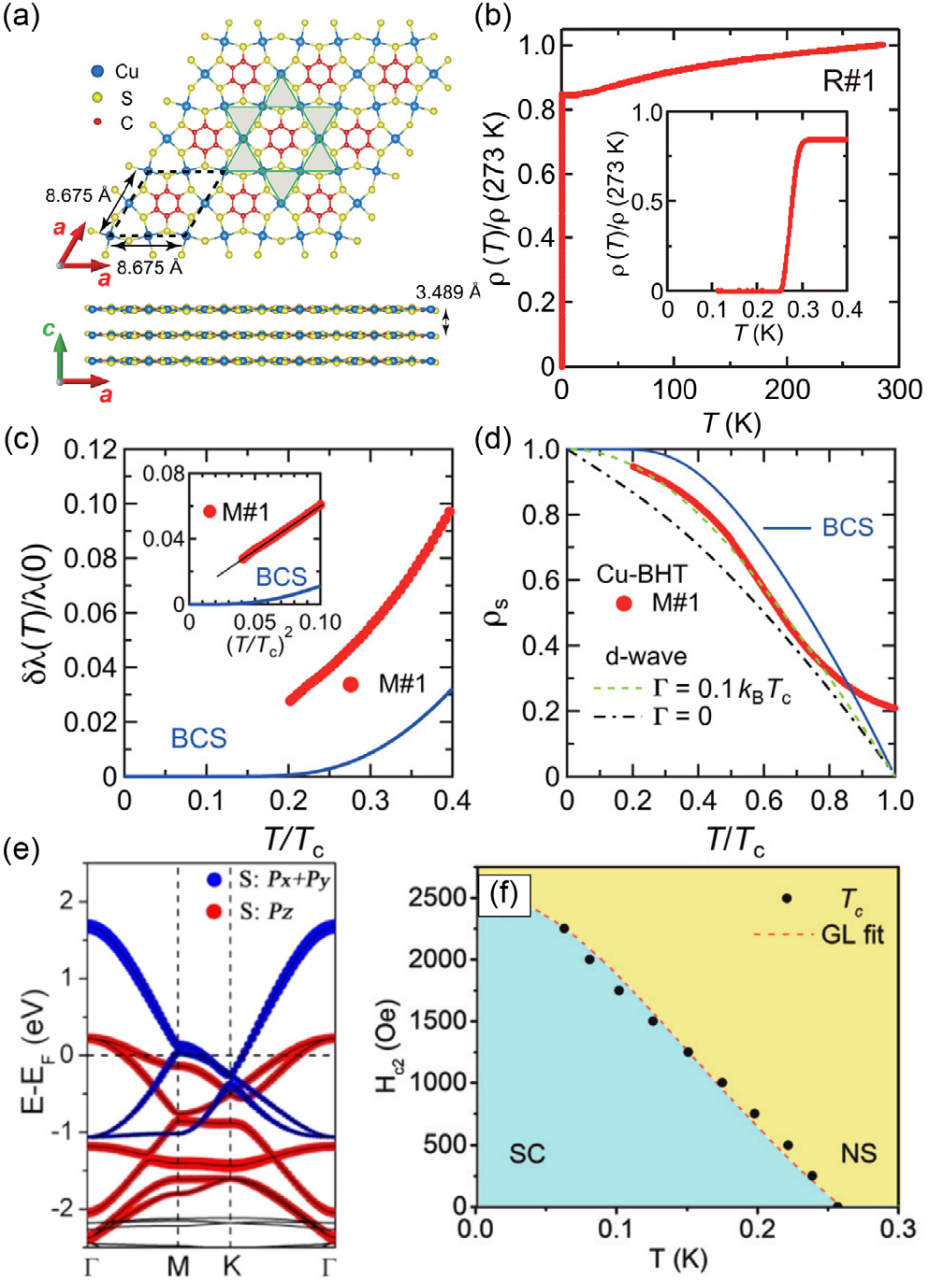
Superconductivity in the highly conductive 2D‐MOF Cu‐BHT. a) Kagome‐type crystal structure. b) The resistivity of the sample, with a sudden drop at the superconducting phase transition around 0.25 K. c,d) The measurements of magnetic penetration depth and normalized superfluid density. e) The band stucture, calculated using density functional theory. f) Phase diagram of Cu‐BHT plotted against temperature and applied magnetic field. (a)–(d) Reproduced with permission.^[^ ^161^, ^163^ ^]^ Copyright 2021, American Association for the Advancement of Science's. (e) Reproduced with permission.^[^ ^161^ ^]^ Copyright 2017, American Chemical Society. (f) Reproduced with permission.^[^ ^162^ ^]^ Copyright 2017, Wiley.^[^ ^162^, ^163^ ^]^

The term heavy fermion describes a system where the kinetic energy of the electronic excitations is heavily suppressed, while particle–particle interactions become dominant. Hence, MOFs which typically show narrow bands are likely to host unconventional superconductivity, given they are metallic in the normal state. One example of a highly conductive 2D MOF is Cu‐BHT ([Cu_3_(C_6_S_6_)]_*n*_), first synthesized by Huang et al.^[^ ^160^ ^]^ The material shows a Kagome structure, shown in **Figure** **10**a. Zhang et al. studied Cu‐BHT using density functional theory. In their calculations, they found a metallic band structure (Figure 10e) where two blocks of bands cross the Fermi level *E*_F_. One block is primarily composed of in‐plane orbitals (mainly S‐*p*_*x*_ and S‐*p*_*y*_), while the other block of bands is primarily composed of out‐of‐plane orbitals (mainly S‐*p*_*z*_). While the in‐plane orbitals give rise to a bandwidth of ≈3 eV, the out‐of‐plane orbitals produce a narrow band with a bandwidth <1 eV.

Furthermore, Zhang et al.^[^ ^161^ ^]^ performed a theoretical prediction of superconducting transition temperatures in 2D and 3D Cu‐BHT applying the conventional BCS theory on top of a density functional theory calculation, deriving transition temperatures of $T_{c}=4.43K$ for 2D Cu‐BHT and $T_{c}=1.58K$ for 3D Cu‐BHT. Remarkably, shortly after, the superconductivity in 2D Cu‐BHT could be verified experimentally.^[^ ^162^, ^163^ ^]^ However, the superconducting transition temperature is much smaller, $T_{c}=0.25K$, as can be seen from the resistivity measurement, for example, Figure 10b. Furthermore, measurements of the magnetic penetration depths and superfluid density, Figure 10c,d, respectively, point toward unconventional superconductivity with nodes in the superconducting gap. The breakdown of the superconductivity under a strong magnetic field is shown in Figure 10f and follows the form of a type‐I superconductor.

### 4.2 Quantum Buckling

Under sufficient axial load, a column buckles, a phenomenon applicable even at the nanoscale where quantum effects become significant. This is relevant in the context of mechanical qubits.^[^ ^164^, ^165^ ^]^ In materials like MOFs, mechanical properties are defined by molecular linkers and the MOF topology. Molecular buckling emerges due to chemical composition, crystal load,^[^ ^49^ ^]^ or pressure.^[^ ^166^ ^]^

**Figure 11:**
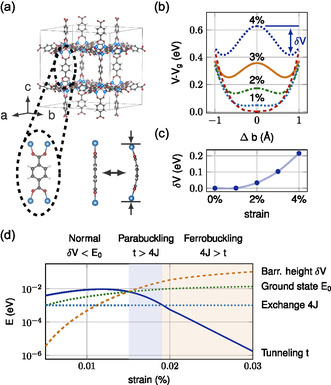
Predicted quantum buckling in MOF‐5. a) Crystal structure of MOF‐5 built of Zn_4_O clusters, coordinated by BDC molecules. b) Energy profile of buckled BDC molecules, forming a double‐well potential with a barrier height *δV*. c) Barrier height against strain along the crystallographic *c*‐direction. d) Buckling phase diagram. Reproduced with permission.^[^ ^64^ ^]^ Copyright 2021, American Chemical Society.

Quantum buckling is predicted to exist in MOFs.^[^ ^64^ ^]^ Consider a linker molecule exhibiting two classical buckling solutions under pressure: left (*L*) and right (*R*) (see **Figure** **11**a for the example of BDC in MOF‐5). These classical solutions refer to the minima in the total energy landscape when the molecule is exposed to uniaxial pressure, which takes the form of a double‐well potential (Figure 11b). The larger the pressure, the deeper the double well, leading to an increase in buckling and barrier width (Figure 11c). In the quantum regime, the classical solutions become quantum states, denoted by |L⟩ and |R⟩. Introducing a finite hopping *t* between these two energetically degenerate states leads to an overlap Hamiltonian.

(19) H=t(|R⟩⟨L|+|L⟩⟨R|)

In matrix form, this Hamiltonian can be expressed as $\langle \alpha |H|\beta \rangle =t\;\sigma_{\alpha \beta }^{x}$, with α,β=L,R and $\sigma_{\alpha ,\beta }^{x}$ the Pauli matrix *x*.

For a lattice of buckled molecules in a MOF, the interactions between buckled molecules can be captured in a transverse field Ising model.^[^ ^64^ ^]^

(20) $$H=-t\sum_{i}\sigma_{i}^{x}-\sum_{ij}J_{ij}\sigma_{i}^{z}\sigma_{j}^{z}$$

Here, *J*_*ij*_ is an exchange energy between molecules *i* and *j* and *σ* ^*z*^ the Pauli matrix *z*. In the nearest‐neighbor limit, with $J_{ij}=J$, the mean‐field limit solution yields a phase diagram, depicted for MOF‐5 in Figure 11d.

This phase diagram illustrates three phases – normal, parabuckling, and ferrobuckling – determined by parameters such as barrier height, exchange energy *J*, tunneling strength *t*, and the molecule's ground state energy. At low strain, no buckling occurs. When the barrier height exceeds ground state energy, quantum states emerge. If tunneling dominates, quantum fluctuations prevent ordered buckling. Otherwise, collective buckling occurs, with a transition temperature of $T_{c}\approx 4J$.

## 5 Rotational Dynamics

The complex crystal structures of MOFs exhibit various intrinsic dynamics, including phonons, magnons, and unconventional degrees of freedom such as molecular rotations and buckling dynamics.^[^ ^167^ ^]^ This shift from static to dynamic structure perception introduces the concept of 4D MOFs.^[^ ^168^, ^169^, ^170^ ^]^

**Figure 12:**
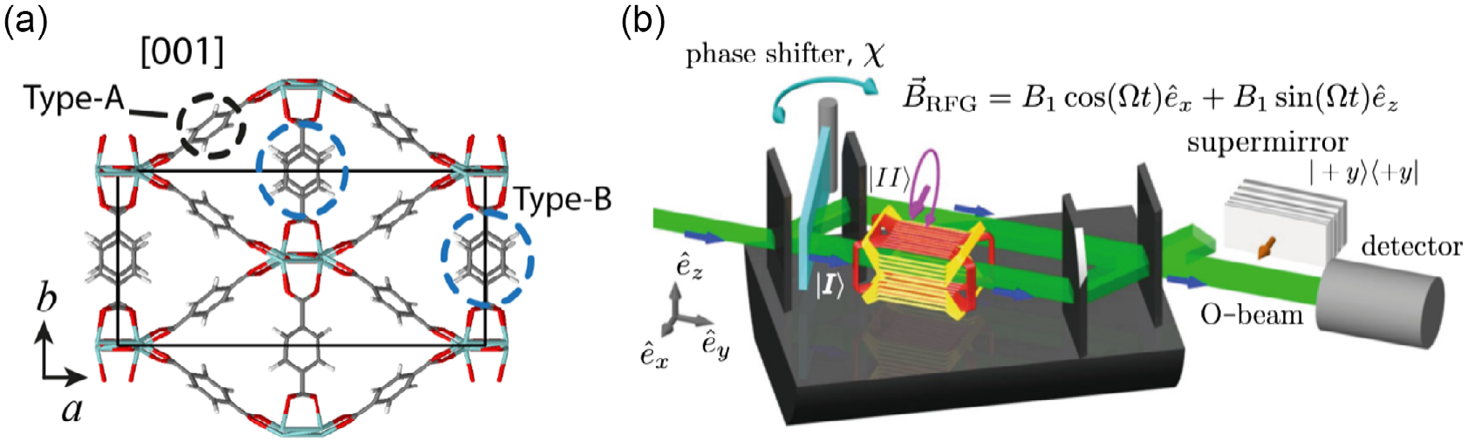
Rotations and inertia. a) Collective rotational modes in MIL‐140A [ZrO(O_2_C‐C_6_H_4_‐CO_2_)].^[^ ^63^ ^]^ b) Neutron interferrometry experiment by Danner et al.^[^ ^192^ ^]^ confirming the spin‐rotation coupling. Comparing (a,b), electrons in the MIL‐140A framework lattice should experience the spin‐rotation coupling in a similar manner. (a) Reproduced with permission.^[^ ^63^ ^]^ Copyright 2017, American Physical Society. (b) Reproduced with permission.^[^ ^192^ ^]^ Copyright 2020, Springer.

Rotation of linker molecules is a significant aspect of MOF dynamics.^[^ ^171^ ^]^ At elevated temperatures or under external stimuli like laser pumping, linker molecules can undergo complete rotations, observed in various MOFs^[^ ^63^, ^172^, ^173^, ^174^, ^175^, ^176^, ^177^, ^178^, ^179^, ^180^ ^]^ (see **Figure** **12**a). This rotational dynamics carries an angular momentum and, as a consequence, couples to the electronic spin. In the case of axial phonons, this coupling is huge, leading to a prominent magnetization.^[^ ^181^, ^182^, ^183^, ^184^, ^185^ ^]^ Translating this effect to MOFs promises the design of highly sensitive field strengths measuring devices and applications in dark matter detection.^[^ ^186^ ^]^ Furthermore, rotational degrees of freedom call for inertial effects, stemming from fictitious forces generated by an accelerating motion relative to a reference frame. While extensively examined in classical^[^ ^187^ ^]^ and quantum mechanics,^[^ ^188^, ^189^ ^]^ inertial effects have recently surfaced as a potential mechanism for coupling linker rotations and electron spin.^[^ ^190^ ^]^

Classically, the Coriolis force on a particle in a rotating frame is ${\vec{F}}_{\text{Coriolis}}=2\vec{p}\times \vec{\omega }$. Multiplying this force by position $\vec{r}$ yields the energy $E=\vec{r}\cdot {\vec{F}}_{\text{Coriolis}}=\vec{\omega }\cdot \vec{L}$, where $\vec{L}$ is the electron's angular momentum. In quantum mechanics, this leads to the spin‐rotation term in the Hamiltonian as $H=\vec{\omega }\cdot \vec{J}$, with the total angular momentum $\vec{J}=\vec{L}\;+\;\vec{S}$ combining orbital angular momentum $\vec{L}$ and spin $\vec{S}$.

On a more rigorous level, inertial effects’ coupling terms to the Hamiltonian can be derived from the Dirac equation. In the nonrelativistic limit, the Dirac equation naturally generates spin and other relativistic corrections, like spin‐orbit interaction or the Darwin term. This formulation in an accelerating frame results in spin and orbital degrees of freedom coupling to inertial forces.^[^ ^189^, ^190^ ^]^ Particularly, for ideal circular ion motion and in the nonrelativistic limit, the spin‐rotation coupling, akin to the Coriolis force, arises along with three additional terms due to the centrifugal force: coupling via the centrifugal field, a redshift component, and an inertial Rashba‐type spin‐orbit interaction (see **Table** **2**).

**Table 2:** Coupling of molecular rotations to the electron, according to ref. [190]. $\vec{S}$, $\vec{J}$, and $\vec{p}$ are the electron spin, total angular momentum, and momentum operators. ${\vec{F}}_{\text{Cent}\text{.}}$ is the centrifugal force ${\vec{F}}_{\text{Cent}\text{.}}=m\omega^{2}\vec{R}$, with $\vec{R}$ the radius circumscribed by the rotation. $\gamma ={\left(1-\frac{{\vec{R}}^{2}\omega^{2}}{c^{2}}\right)}^{-\frac{1}{2}}\approx 1$ is the Lorentz factor.

Spin‐rotation coupling	$\vec{\omega }\cdot \vec{J}$
Centrifugal field coupling	$\gamma^{2}{\vec{F}}_{\text{Cent}\text{.}}\cdot \vec{r}$
Centrifugal spin‐orbit coupling	$\frac{\gamma^{2}}{2m^{2}c^{2}}{\vec{F}}_{\text{Cent}\text{.}}\cdot \left(\vec{S}\times \vec{p}\right)$
Centrifugal redshift	$\frac{\gamma^{2}}{2m^{2}c^{2}}\vec{p}\left({\vec{F}}_{\text{Cent}\text{.}}\cdot \vec{r}\right)\vec{p}$

Inertial effects on quantum systems have been experimentally verified, notably in neutron interferometry. Werner et al.^[^ ^191^ ^]^ measured Earth's rotation effect on a neutron beam's phase using interferometry in 1979. More recently, Danner et al.^[^ ^192^ ^]^ demonstrated spin‐rotation coupling in neutron interferometry (Figure 12), guiding one beam through a rotating magnetic field. While Werner's experiment operated at 1/day≈μHz, Danner et al. worked with frequencies in the kHz range. However, MOF rotations can reach GHz…THz, up to 18 orders of magnitude higher than Earth's rotation. Comparing spin‐rotation coupling $\vec{\omega }\cdot \vec{S}$ to the Zeeman term $\frac{\mu_{B}}{\hbar }\vec{B}\cdot \vec{S}$, a 1 THz rotation corresponds to a ≈10 T magnetic field. Consequently, depending on spin and rotation frequency, level shifts occur in the meV range, detectable with optical experiments. In Figure 12 we compare the neutron interferrometer setup by Danner et al.^[^ ^192^ ^]^ with emergent linker rotation in MIL‐140 A.^[^ ^63^ ^]^ We conclude that electronic levels in the MOF should be strongly affected by the linker dynamics.

## 6 Outlook

MOFs are a promising material class for the design of quantum phenomena. To date, a huge variety of MOFs can be readily synthesized. Their structural properties can be adjusted and designed by varying IBUs and linkers, both in 2D and 3D.

However, synthesizing MOFs with coherent quantum states presents significant challenges, primarily due to difficulties in achieving high purity and low defect crystals. Strategies such as using competing reagents to slow crystal growth and enhance purity are necessary.^[^ ^53^ ^]^ While CVD has been explored for high‐quality MOFs, its application remains limited.^[^ ^54^, ^55^ ^]^ Precision techniques like scanning probe manipulation offer controlled growth and high purity but lack scalability.^[^ ^56^ ^]^ Additionally, controlling buckling and mechanical instabilities in MOFs is challenging, with strategies like promoting *π*–*π* interactions within the structure showing promise.^[^ ^57^ ^]^

Another challenge for electronic phases in quantum materials research lies in achieving (semi)conductivity in MOFs.^[^ ^32^, ^105^ ^]^ Generally, due to suppressed hopping strengths, MOFs show strong correlation effects which can be altered by the MOF topology. Furthermore, the lability of the MOF building blocks affects the formation of defects and disorder, through the synthesis process,^[^ ^60^ ^]^ hindering functionality and conductivity. Incorporating unconventional degrees of freedom into the equation allows the design of totally new phases of matter, such as collective quantum buckling. Also, the softness of the MOFs naturally introduces time into the concept as MOF structures often cannot be regarded as static objects, but instead strongly evolve in time. Incorporating collective rotational degrees of freedom, for example, will influence the electronic properties, among others due to strong spin‐phonon coupling and inertial effects. Intermolecular interactions, such as H‐bonding and *π*–*π* interactions, can be tailored to control such a rotational dynamics. While significant attention has been paid on realizing Hall devices based on topological electrons in 2D MOFs, the general field of discussing MOFs as quantum materials is only at the beginning. Given the enormous potential of MOFs and their breakthrough applications in chemistry, we strongly believe that these materials can lead to a paradigm change in the field of quantum materials in the upcoming decade.

## Conflict of Interest

The authors declare no conflict of interest.

## References

[1] B. Keimer , J. Moore , Nat. Phys. 2017, 13, 1045.

[2] J. Linder , A. V. Balatsky , Rev. Mod. Phys. 2019, 91, 045005.

[3] M. P. Zaletel , M. Lukin , C. Monroe , C. Nayak , F. Wilczek , N. Y. Yao , Rev. Mod. Phys. 2023, 95, 031001.

[4] T. Oka , S. Kitamura , Annu. Rev. Condens. Matter Phys. 2019, 10, 387.

[5] Y. Tokura , M. Kawasaki , N. Nagaosa , Nat. Phys. 2017, 13, 1056.

[6] X.-G. Wen , Science 2019, 363, eaal3099.30792273

[7] W. Brzezicki , J. Phys.: Condens. Matter 2019, 32, 023001. 10.1088/1361-648X/ab448d 31519012

[8] S. Sachdev , Rep. Prog. Phys. 2018, 82, 014001. 30210062 10.1088/1361-6633/aae110

[9] X.-G. Wen , Rev. Mod. Phys. 2017, 89, 041004.

[10] C. Castelnovo , R. Moessner , S. L. Sondhi , Annu. Rev. Condens. Matter Phys. 2012, 3, 35.

[11] A. de la Torre , D. M. Kennes , M. Claassen , S. Gerber , J. W. McIver , M. A. Sentef , Rev. Mod. Phys. 2021, 93, 041002.

[12] M. Heyl , Rep. Prog. Phys. 2018, 81, 054001. 29446351 10.1088/1361-6633/aaaf9a

[13] A. E. Thorarinsdottir , T. D. Harris , Chem. Rev. 2020, 120, 8716. 32045215 10.1021/acs.chemrev.9b00666

[14] M. Kurmoo , Chem. Soc. Rev. 2009, 38, 1353. 19384442 10.1039/b804757j

[15] X. Ni , H. Li , F. Liu , J.-L. Brédas , Mater. Horiz. 2022, 9, 88. 34866138 10.1039/d1mh00935d

[16] W. Jiang , S. Zhang , Z. Wang , F. Liu , T. Low , Nano Lett. 2020, 20, 1959. 32078326 10.1021/acs.nanolett.9b05242

[17] S.-Y. Ding , W. Wang , Chem. Soc. Rev. 2013, 42, 548. 23060270 10.1039/c2cs35072f

[18] H. M. El‐Kaderi , J. R. Hunt , J. L. Mendoza‐Cortés , A. P. Côté , R. E. Taylor , M. O’Keeffe , O. M. Yaghi , Science 2007, 316, 268. 17431178 10.1126/science.1139915

[19] R.-B. Lin , B. Chen , Chem 2022, 8, 2114.

[20] R.-B. Lin , Y. He , P. Li , H. Wang , W. Zhou , B. Chen , Chem. Soc. Rev. 2019, 48, 1362. 30676603 10.1039/c8cs00155c PMC11061856

[21] O. M. Yaghi , G. Li , H. Li , Nature 1995, 378, 703.

[22] S. Kitagawa , R. Kitaura , S.-I. Noro , Angew. Chem., Int. Ed. 2004, 43, 2334. 10.1002/anie.200300610 15114565

[23] H. Li , M. Eddaoudi , T. L. Groy , O. Yaghi , J. Am. Chem. Soc. 1998, 120, 8571.

[24] Q. Li , W. Zhang , O. Š. Miljanić , C.-H. Sue , Y.-L. Zhao , L. Liu , C. B. Knobler , J. F. Stoddart , O. M. Yaghi , Science 2009, 325, 855. 19679809 10.1126/science.1175441

[25] C. A. Trickett , A. Helal , B. A. Al‐Maythalony , Z. H. Yamani , K. E. Cordova , O. M. Yaghi , Nat. Rev. Mater. 2017, 2, 17045.

[26] Z. Chen , P. Li , X. Zhang , P. Li , M. C. Wasson , T. Islamoglu , J. F. Stoddart , O. K. Farha , J. Am. Chem. Soc. 2019, 141, 2900. 30735359 10.1021/jacs.8b13710

[27] K.-G. Liu , Z. Sharifzadeh , F. Rouhani , M. Ghorbanloo , A. Morsali , Coord. Chem. Rev. 2021, 436, 213827.

[28] J. Duan , W. Jin , S. Kitagawa , Coord. Chem. Rev. 2017, 332, 48.

[29] N. S. Bobbitt , M. L. Mendonca , A. J. Howarth , T. Islamoglu , J. T. Hupp , O. K. Farha , R. Q. Snurr , Chem. Soc. Rev. 2017, 46, 3357. 28345694 10.1039/c7cs00108h

[30] R.-B. Lin , S. Xiang , H. Xing , W. Zhou , B. Chen , Coord. Chem. Rev. 2019, 378, 87. 10.1016/j.ccr.2017.09.027 PMC11467812 39398898

[31] T. Zhang , W. Lin , Chem. Soc. Rev. 2014, 43, 5982. 24769551 10.1039/c4cs00103f

[32] D. Sheberla , J. C. Bachman , J. S. Elias , C.-J. Sun , Y. Shao‐Horn , M. Dincă , Nat. Mater. 2017, 16, 220. 27723738 10.1038/nmat4766

[33] D. Feng , T. Lei , M. R. Lukatskaya , J. Park , Z. Huang , M. Lee , L. Shaw , S. Chen , A. A. Yakovenko , A. Kulkarni , J. Xiao , Nat. Energy 2018, 3, 30.

[34] J. Park , M. Lee , D. Feng , Z. Huang , A. C. Hinckley , A. Yakovenko , X. Zou , Y. Cui , Z. Bao , J. Am. Chem. Soc. 2018, 140, 10315. 30041519 10.1021/jacs.8b06020

[35] G. Chen , L. B. Gee , W. Xu , Y. Zhu , J. S. Lezama‐Pacheco , Z. Huang , Z. Li , J. T. Babicz Jr , S. Choudhury , T.-H. Chang , E. Reed , J. Am. Chem. Soc. 2020, 142, 21243. 33315385 10.1021/jacs.0c09379 PMC7888195

[36] J. Park , A. C. Hinckley , Z. Huang , G. Chen , A. A. Yakovenko , X. Zou , Z. Bao , J. Am. Chem. Soc. 2020, 142, 20531. 33226798 10.1021/jacs.0c09573

[37] J. Della Rocca , D. Liu , W. Lin , Acc. Chem. Res. 2011, 44, 957. 21648429 10.1021/ar200028a PMC3777245

[38] P. Horcajada , R. Gref , T. Baati , P. K. Allan , G. Maurin , P. Couvreur , G. Férey , R. E. Morris , C. Serre , Chem. Rev. 2012, 112, 1232. 22168547 10.1021/cr200256v

[39] L. E. Kreno , K. Leong , O. K. Farha , M. Allendorf , R. P. Van Duyne , J. T. Hupp , Chem. Rev. 2012, 112, 1105. 22070233 10.1021/cr200324t

[40] Z. Hu , B. J. Deibert , J. Li , Chem. Soc. Rev. 2014, 43, 5815. 24577142 10.1039/c4cs00010b

[41] K. Lu , T. Aung , N. Guo , R. Weichselbaum , W. Lin , Adv. Mater. 2018, 30, 1707634. 10.1002/adma.201707634 PMC6586248 29971835

[42] Y. Cui , Y. Yue , G. Qian , B. Chen , Chem. Rev. 2012, 112, 1126. 21688849 10.1021/cr200101d

[43] J. Lee , O. K. Farha , J. Roberts , K. A. Scheidt , S. T. Nguyen , J. T. Hupp , Chem. Soc. Rev. 2009, 38, 1450. 19384447 10.1039/b807080f

[44] M. Yoon , R. Srirambalaji , K. Kim , Chem. Rev. 2012, 112, 1196. 22084838 10.1021/cr2003147

[45] X. Wang , X. Han , J. Zhang , X. Wu , Y. Liu , Y. Cui , J. Am. Chem. Soc. 2016, 138, 12332. 27618953 10.1021/jacs.6b07714

[46] F. J. Carmona , C. R. Maldonado , S. Ikemura , C. C. Romao , Z. Huang , H. Xu , X. Zou , S. Kitagawa , S. Furukawa , E. Barea , ACS Appl. Mater. Interfaces 2018, 10, 31158. 30152684 10.1021/acsami.8b11758

[47] S. Roy , Z. Huang , A. Bhunia , A. Castner , A. K. Gupta , X. Zou , S. Ott , J. Am. Chem. Soc. 2019, 141, 15942. 31508946 10.1021/jacs.9b07084 PMC6803166

[48] M. Eddaoudi , J. Kim , N. Rosi , D. Vodak , J. Wachter , M. O’Keeffe , O. M. Yaghi , Science 2002, 295, 469. 11799235 10.1126/science.1067208

[49] A. Schneemann , V. Bon , I. Schwedler , I. Senkovska , S. Kaskel , R. A. Fischer , Chem. Soc. Rev. 2014, 43, 6062. 24875583 10.1039/c4cs00101j

[50] S.-H. Lo , L. Feng , K. Tan , Z. Huang , S. Yuan , K.-Y. Wang , B.-H. Li , W.-L. Liu , G. S. Day , S. Tao , C. C. Yang , Nat. Chem. 2020, 12, 90. 31792388 10.1038/s41557-019-0364-0

[51] S. Yuan , L. Huang , Z. Huang , D. Sun , J.-S. Qin , L. Feng , J. Li , X. Zou , T. Cagin , H.-C. Zhou , J. Am. Chem. Soc. 2020, 142, 4732. 32058715 10.1021/jacs.9b13072

[52] M. O’Keeffe , M. A. Peskov , S. J. Ramsden , O. M. Yaghi , Acc. Chem. Res. 2008, 41, 1782, PMID: 18834152. 18834152 10.1021/ar800124u

[53] R. S. Forgan , Chem. Sci. 2020, 11, 4546. 34122913 10.1039/d0sc01356k PMC8159241

[54] I. Stassen , M. Styles , G. Grenci , H. V. Gorp , W. Vanderlinden , S. D. Feyter , P. Falcaro , D. D. Vos , P. Vereecken , R. Ameloot , Nat. Mater. 2016, 15, 304. 26657328 10.1038/nmat4509

[55] M. Choe , J. Y. Koo , I. Park , H. Ohtsu , J. H. Shim , H. C. Choi , S. S. Park , J. Am. Chem. Soc. 2022, 144, 16726. 36095292 10.1021/jacs.2c07135

[56] Q. Zhong , A. Ihle , S. Ahles , H. A. Wegner , A. Schirmeisen , D. Ebeling , Nat. Chem. 2021, 13, 1133. 34475530 10.1038/s41557-021-00773-4 PMC8550974

[57] L. Samperisi , A. Jaworski , G. Kaur , K. P. Lillerud , X. Zou , Z. Huang , J. Am. Chem. Soc. 2021, 143, 17947. 34695352 10.1021/jacs.1c08354 PMC8569804

[58] C. Serre , F. Millange , C. Thouvenot , M. Nogues , G. Marsolier , D. Loüer , G. Férey , J. Am. Chem. Soc. 2002, 124, 13519. 12418906 10.1021/ja0276974

[59] S. Horike , S. Shimomura , S. Kitagawa , Nat. Chem. 2009, 1, 695. 21124356 10.1038/nchem.444

[60] R. E. Morris , L. Brammer , Chem. Soc. Rev. 2017, 46, 5444. 28795752 10.1039/c7cs00187h

[61] F. Bigdeli , C. T. Lollar , A. Morsali , H.-C. Zhou , Angew. Chem., Int. Ed. 2020, 59, 4652. 10.1002/anie.201900666 31134738

[62] D. Fairen‐Jimenez , S. A. Moggach , M. T. Wharmby , P. A. Wright , S. Parsons , T. Düren , J. Am. Chem. Soc. 2011, 133, 8900. 21553843 10.1021/ja202154j

[63] M. R. Ryder , B. Van de Voorde , B. Civalleri , T. D. Bennett , S. Mukhopadhyay , G. Cinque , F. FernandezAlonso , D. De Vos , S. Rudić , J.-C. Tan , Phys. Rev. Lett. 2017, 118, 255502. 28696751 10.1103/PhysRevLett.118.255502

[64] R. M. Geilhufe , Nano Lett. 2021, 21, 10341. 34881896 10.1021/acs.nanolett.1c03579 PMC8704192

[65] R. Dong , M. Pfeffermann , H. Liang , Z. Zheng , X. Zhu , J. Zhang , X. Feng , Angew. Chem., Int. Ed. 2015, 54, 12058. 10.1002/anie.201506048 26306686

[66] T. Kambe , R. Sakamoto , K. Hoshiko , K. Takada , M. Miyachi , J.-H. Ryu , S. Sasaki , J. Kim , K. Nakazato , M. Takata , H. Nishihara , J. Am. Chem. Soc. 2013, 135, 2462. 23360513 10.1021/ja312380b

[67] N. A. Vermeulen , O. Karagiaridi , A. A. Sarjeant , C. L. Stern , J. T. Hupp , O. K. Farha , J. F. Stoddart , J. Am. Chem. Soc. 2013, 135, 14916. 24047342 10.1021/ja407333q

[68] S. Takaishi , E. J. DeMarco , M. J. Pellin , O. K. Farha , J. T. Hupp , Chem. Sci. 2013, 4, 1509.

[69] J.-S. M. Lee , T. Kurihara , S. Horike , Chem. Mater. 2020, 32, 7694.

[70] T. Yang , T. Willhammar , H. Xu , X. Zou , Z. Huang , Nat. Protoc. 2022, 17, 2389. 35896741 10.1038/s41596-022-00720-8

[71] K. V. Klitzing , G. Dorda , M. Pepper , Phys. Rev. Lett. 1980, 45, 494.

[72] R. B. Laughlin , Phys. Rev. B 1981, 23, 5632.

[73] B. I. Halperin , Phys. Rev. B 1982, 25, 2185.

[74] D. J. Thouless , M. Kohmoto , M. P. Nightingale , M. den Nijs , Phys. Rev. Lett. 1982, 49, 405.

[75] B. Simon , Phys. Rev. Lett. 1983, 51, 2167.

[76] F. D. M. Haldane , Phys. Rev. Lett. 1988, 61, 2015. 10038961 10.1103/PhysRevLett.61.2015

[77] K. S. Novoselov , A. K. Geim , S. V. Morozov , D. Jiang , M. I. Katsnelson , I. V. Grigorieva , S. Dubonos , A. Firsov , Nature 2005, 438, 197. 16281030 10.1038/nature04233

[78] C. L. Kane , E. J. Mele , Phys. Rev. Lett. 2005, 95, 226801. 16384250 10.1103/PhysRevLett.95.226801

[79] B. A. Bernevig , T. L. Hughes , S.-C. Zhang , Science 2006, 314, 1757. 17170299 10.1126/science.1133734

[80] L. Fu , C. L. Kane , E. J. Mele , Phys. Rev. Lett. 2007, 98, 106803. 17358555 10.1103/PhysRevLett.98.106803

[81] J. E. Moore , L. Balents , Phys. Rev. B 2007, 75, 121306.

[82] R. Roy , Phys. Rev. B 2009, 79, 195322.

[83] D. Hsieh , D. Qian , L. Wray , Y. Xia , Y. S. Hor , R. J. Cava , M. Z. Hasan , Nature 2008, 452, 970. 18432240 10.1038/nature06843

[84] Y. Xia , D. Qian , D. Hsieh , L. Wray , A. Pal , H. Lin , A. Bansil , D. Grauer , Y. S. Hor , R. J. Cava , M. Z. Hasan , Nat. Phys. 2009, 5, 398. 10.1103/PhysRevLett.103.146401 19905585

[85] Y. Chen , J. G. Analytis , J.-H. Chu , Z. Liu , S.-K. Mo , X.-L. Qi , H. Zhang , D. Lu , X. Dai , Z. Fang , S. C. Zhang , Science 2009, 325, 178. 19520912 10.1126/science.1173034

[86] D. Hsieh , Y. Xia , D. Qian , L. Wray , F. Meier , J. H. Dil , J. Osterwalder , L. Patthey , A. V. Fedorov , H. Lin , A. Bansil , D. Grauer , Y. S. Hor , R. J. Cava , M. Z. Hasan , Phys. Rev. Lett. 2009, 103, 146401. 19905585 10.1103/PhysRevLett.103.146401

[87] P. Dziawa , B. Kowalski , K. Dybko , R. Buczko , A. Szczerbakow , M. Szot , E. Lusakowska , T. Balasubramanian , B. M. Wojek , M. Berntsen , O. Tjernberg , Nat. Mater. 2012, 11, 1023. 23023551 10.1038/nmat3449

[88] M. Z. Hasan , C. L. Kane , Rev. Mod. Phys. 2010, 82, 3045.

[89] Y. Tokura , K. Yasuda , A. Tsukazaki , Nat. Rev. Phys. 2019, 1, 126.

[90] K. S. Novoselov , A. Mishchenko , A. Carvalho , H. C. Neto , Science 2016, 353, aac9439. 27471306 10.1126/science.aac9439

[91] F. Miao , S.-J. Liang , B. Cheng , npj Quantum Mater. 2021, 6, 59.

[92] S. K. Behura , A. Miranda , S. Nayak , K. Johnson , P. Das , N. R. Pradhan , Emergent Mater. 2021, 4, 813.

[93] X. Sun , M. Suriyage , A. R. Khan , M. Gao , J. Zhao , B. Liu , M. M. Hasan , S. Rahman , R. Chen , P. K. Lam , Y. Lu , Chem. Rev. 2024, 124, 1992. 38335114 10.1021/acs.chemrev.3c00627

[94] X. Wang , K. Du , Y. Y. F. Liu , P. Hu , J. Zhang , Q. Zhang , M. H. S. Owen , X. Lu , C. K. Gan , P. Sengupta , C. Kloc , Q. Xiong , 2D Mater. 2016, 3, 031009.

[95] J.-U. Lee , S. Lee , J. H. Ryoo , S. Kang , T. Y. Kim , P. Kim , C.-H. Park , J.-G. Park , H. Cheong , Nano Lett. 2016, 16, 7433. 27960508 10.1021/acs.nanolett.6b03052

[96] B. Huang , G. Clark , E. Navarro‐Moratalla , D. R. Klein , R. Cheng , K. L. Seyler , D. Zhong , E. Schmidgall , M. A. McGuire , D. H. Cobden , W. Yao , D. Xiao , P. JarilloHerrero , X. Xu , Nature 2017, 546, 270. 28593970 10.1038/nature22391

[97] C. Gong , L. Li , Z. Li , H. Ji , A. Stern , Y. Xia , T. Cao , W. Bao , C. Wang , Y. Wang , Z. Q. Qiu , R. J. Cava , S. G. Louie , J. Xia , X. Zhang , Nature 2017, 546, 265. 28445468 10.1038/nature22060

[98] J. López‐Cabrelles , S. Man∼as‐Valero , I. J. VitóricaYrezábal , M. Šiškins , M. Lee , P. G. Steeneken , H. S. J. van der Zant , G. M. Espallargas , E. Coronado , J. Am. Chem. Soc. 2021, 143, 18502. 34723487 10.1021/jacs.1c07802 PMC8587609

[99] M. Blei , J. L. Lado , Q. Song , D. Dey , O. Erten , V. Pardo , R. Comin , S. Tongay , A. S. Botana , Appl. Phys. Rev. 2021, 8, 021301.

[100] G. D. Nguyen , H.-Z. Tsai , A. A. Omrani , T. Marangoni , M. Wu , D. J. Rizzo , G. F. Rodgers , R. R. Cloke , R. A. Durr , Y. Sakai , F. Liou , A. S. Aikawa , J. R. Chelikowsky , S. G. Louie , F. R. Fischer , M. F. Crommie , Nat. Nanotechnol. 2017, 12, 1077. 28945240 10.1038/nnano.2017.155

[101] D. J. Rizzo , G. Veber , T. Cao , C. Bronner , T. Chen , F. Zhao , H. Rodriguez , S. G. Louie , M. F. Crommie , F. R. Fischer , Nature 2018, 560, 204. 30089918 10.1038/s41586-018-0376-8

[102] E. C. Regan , D. Wang , C. Jin , M. I. B. Utama , B. Gao , X. Wei , S. Zhao , W. Zhao , Z. Zhang , K. Yumigeta , M. Blei , J. D. Carlström , K. Watanabe , T. Taniguchi , S. Tongay , M. Crommie , A. Zettl , F. Wang , Nature 2020, 579, 359. 32188951 10.1038/s41586-020-2092-4

[103] S. Tang , C. Zhang , D. Wong , Z. Pedramrazi , H.-Z. Tsai , C. Jia , B. Moritz , M. Claassen , H. Ryu , S. Kahn , J. Jiang , H. Yan , M. Hashimoto , D. Lu , R. G. Moore , C.-C. Hwang , C. Hwang , Z. Hussain , Y. Chen , M. M. Ugeda , Z. Liu , X. Xie , T. P. Devereaux , M. F. Crommie , S.-K. Mo , Z.-X. Shen , Nat. Phys. 2017, 13, 683.

[104] Y. Zhang , T.-T. Tang , C. Girit , Z. Hao , M. C. Martin , A. Zettl , M. F. Crommie , Y. R. Shen , F. Wang , Nature 2009, 459, 820. 19516337 10.1038/nature08105

[105] C.-Z. Chang , C.-X. Liu , A. H. MacDonald , Rev. Mod. Phys. 2023, 95, 011002.

[106] Z. Wang , Z. Liu , F. Liu , Nat. Commun. 2013, 4, 1471. 23403572 10.1038/ncomms2451

[107] Z. Wang , N. Su , F. Liu , Nano Lett. 2013, 13, 2842. 23678979 10.1021/nl401147u

[108] Z. F. Wang , Z. Liu , F. Liu , Phys. Rev. Lett. 2013, 110, 196801. 23705732 10.1103/PhysRevLett.110.196801

[109] Z. Liu , Z.-F. Wang , J.-W. Mei , Y.-S. Wu , F. Liu , Phys. Rev. Lett. 2013, 110, 106804. 23521279 10.1103/PhysRevLett.110.106804

[110] M. A. Springer , T.-J. Liu , A. Kuc , T. Heine , Chem. Soc. Rev. 2020, 49, 2007. 32206766 10.1039/c9cs00893d

[111] W. Jiang , X. Ni , F. Liu , Acc. Chem. Res. 2021, 54, 416. 33400497 10.1021/acs.accounts.0c00652

[112] R. Fan , L. Sun , X. Shao , Y. Li , M. Zhao , ChemPhysMater 2022, 2, 30.

[113] C.-H. Hsu , Z.-Q. Huang , G. M. Macam , F.-C. Chuang , L. Huang , Appl. Phys. Lett. 2018, 113, 233301.

[114] B. Zhao , J. Zhang , W. Feng , Y. Yao , Z. Yang , Phys. Rev. B 2014, 90, 201403.

[115] D. Sheberla , L. Sun , M. A. Blood‐Forsythe , S. Er , C. R. Wade , C. K. Brozek , A. Aspuru‐Guzik , M. Dincă , J. Am. Chem. Soc. 2014, 136, 8859. 24750124 10.1021/ja502765n

[116] Z. Gao , C.-H. Hsu , J. Liu , F.-C. Chuang , R. Zhang , B. Xia , H. Xu , L. Huang , Q. Jin , P. N. Liu , N. Lin , Nanoscale 2019, 11, 878. 30604812 10.1039/c8nr08477g

[117] Z. Gao , Y. Gao , M. Hua , J. Liu , L. Huang , N. Lin , J. Phys. Chem. C 2020, 124, 27017.

[118] Y. Yang , Z. Xu , L. Sheng , B. Wang , D. Y. Xing , D. N. Sheng , Phys. Rev. Lett. 2011, 107, 066602. 21902351 10.1103/PhysRevLett.107.066602

[119] V. N. Kotov , B. Uchoa , V. M. Pereira , F. Guinea , A. H. Castro Neto , Rev. Mod. Phys. 2012, 84, 1067.

[120] J. González , F. Guinea , M. Vozmediano , Nucl. Phys. B 1994, 424, 595.

[121] E. V. Gorbar , V. P. Gusynin , V. A. Miransky , I. A. Shovkovy , Phys. Rev. B 2002, 66, 045108.

[122] I. F. Herbut , V. Juričić , O. Vafek , Phys. Rev. B 2009, 80, 075432. 10.1103/PhysRevLett.100.046403 18352311

[123] V. Juričić , I. F. Herbut , G. W. Semenoff , Phys. Rev. B 2009, 80, 081405.

[124] D. Khveshchenko , J. Phys.: Condens. Matter 2009, 21, 075303. 21817324 10.1088/0953-8984/21/7/075303

[125] O. V. Gamayun , E. V. Gorbar , V. P. Gusynin , Phys. Rev. B 2010, 81, 075429.

[126] J. González , J. High Energy Phys. 2015, 2015, 190.

[127] P. O. Sukhachov , V. Juričić , A. V. Balatsky , Phys. Rev. B 2019, 100, 180502.

[128] H. Wei , S.-P. Chao , V. Aji , Phys. Rev. Lett. 2012, 109, 196403. 23215410 10.1103/PhysRevLett.109.196403

[129] H. Wei , S.-P. Chao , V. Aji , Phys. Rev. B 2014, 89, 014506.

[130] B. Roy , P. Goswami , V. Juričić , Phys. Rev. B 2017, 95, 201102.

[131] V. Juričić , I. S. Landea , R. Soto‐Garrido , J. High Energy Phys. 2020, 2020, 52.

[132] A. Balatsky , Philos. Mag. Lett. 1993, 68, 251.

[133] R. E. Throckmorton , J. Hofmann , E. Barnes , S. Das Sarma , Phys. Rev. B 2015, 92, 115101.

[134] Y. Nambu , G. Jona‐Lasinio , Phys. Rev. 1961, 122, 345.

[135] T.-D. Lee , Phys. Rep. 1974, 9, 143.

[136] Y. H. Budnikova , Dalton Trans. 2020, 49, 12483. 32756705 10.1039/d0dt01741h

[137] C. Xu , R. Fang , R. Luque , L. Chen , Y. Li , Coord. Chem. Rev. 2019, 388, 268.

[138] V. Pascanu , G. González Miera , A. K. Inge , B. Martín‐Matute , Am. Chem. Soc. 2019, 141, 7223. 10.1021/jacs.9b00733 30974060

[139] L. S. Xie , G. Skorupskii , M. Dinca , Chem. Rev. 2020, 120, 8536. 32275412 10.1021/acs.chemrev.9b00766 PMC7453401

[140] H. Huang , Y. Zhao , Y. Bai , F. Li , Y. Zhang , Y. Chen , Adv. Sci. 2020, 7, 2000012. 10.1002/advs.202000012 PMC7201256 32382489

[141] Y.-C. Zhou , W.-W. Dong , M.-Y. Jiang , Y.-P. Wu , D.-S. Li , Z.-F. Tian , J. Zhao , J. Solid State Chem. 2019, 279, 120929.

[142] V. Khrizanforova , R. Shekurov , V. Miluykov , M. Khrizanforov , V. Bon , S. Kaskel , A. Gubaidullin , O. Sinyashin , Y. Budnikova , Dalton Trans. 2020, 49, 2794. 32073068 10.1039/c9dt04834k

[143] Y.-S. Li , J.-W. Yi , J.-H. Wei , Y.-P. Wu , B. Li , S. Liu , C. Jiang , H.-G. Yu , D.-S. Li , J. Solid State Chem. 2020, 281, 121052.

[144] R. Xie , T. Zhang , H. Weng , G.-L. Chai , Small Sci. 2022, 2, 2100106. 10.1002/smsc.202100106 PMC11935991 40212671

[145] G. Li , C. Felser , Appl. Phys. Lett. 2020, 116, 070501.

[146] J. Li , J. Wu , S. Park , M. Sasase , T.-N. Ye , Y. Lu , M. Miyazaki , T. Yokoyama , T. Tada , M. Kitano , H. Hosono , Sci. Adv. 2023, 9, 38. 10.1126/sciadv.adh9104 PMC10516497 37738353

[147] R. J. Katz , Y. Zhu , Z. Mao , R. E. Schaak , ChemCatChem 2022, 14, e202101714.

[148] V. L. Ginzburg , L. D. Landau , Sov. Phys. JETP 1950, 7, 1064.

[149] J. Bardeen , L. N. Cooper , J. R. Schrieffer , Phys. Rev. 1957, 108, 1175.

[150] L. P. Gor'kov , Sov. Phys. JETP 1959, 9, 1364.

[151] E. Maxwell , Phys. Rev. 1950, 78, 477.

[152] F. Steglich , J. Aarts , C. D. Bredl , W. Lieke , D. Meschede , W. Franz , H. Schäfer , Phys. Rev. Lett. 1979, 43, 1892.

[153] D. Jérome , A. Mazaud , M. Ribault , K. Bechgaard , J. Phys. Lett. 1980, 41, 95.

[154] J. G. Bednorz , K. A. Müller , Z. Phys. B: Condens. Matter 1986, 64, 189.

[155] D. J. Scalapino , E. Loh , J. E. Hirsch , Phys. Rev. B 1987, 35, 6694. 10.1103/physrevb.35.6694 9940917

[156] J. R. Schrieffer , X. G. Wen , S. C. Zhang , Phys. Rev. B 1989, 39, 11663. 10.1103/physrevb.39.11663 9947998

[157] P. Monthoux , A. V. Balatsky , D. Pines , Phys. Rev. Lett. 1991, 67, 3448. 10044736 10.1103/PhysRevLett.67.3448

[158] M. Sigrist , K. Ueda , Rev. Mod. Phys. 1991, 63, 239.

[159] A. V. Balatsky , I. Vekhter , J.-X. Zhu , Rev. Mod. Phys. 2006, 78, 373.

[160] X. Huang , P. Sheng , Z. Tu , F. Zhang , J. Wang , H. Geng , Y. Zou , C.-A. Di , Y. Yi , Y. Sun , W. Xu , Nat. Commun. 2015, 6, 7408. 26074272 10.1038/ncomms8408 PMC4490364

[161] X. Zhang , Y. Zhou , B. Cui , M. Zhao , F. Liu , Nano Lett. 2017, 17, 6166. 28898086 10.1021/acs.nanolett.7b02795

[162] X. Huang , S. Zhang , L. Liu , L. Yu , G. Chen , W. Xu , D. Zhu , Angew. Chem., Int. Ed. 2018, 57, 146. 10.1002/anie.201707568 29160950

[163] T. Takenaka , K. Ishihara , M. Roppongi , Y. Miao , Y. Mizukami , T. Makita , J. Tsurumi , S. Watanabe , J. Takeya , M. Yamashita , K. Torizuka , Sci. Adv. 2021, 7, eabf3996. 33731356 10.1126/sciadv.abf3996 PMC7968839

[164] S. Savel'ev , X. Hu , F. Nori , New J. Phys. 2006, 8, 105.

[165] S. Savel'ev , A. L. Rakhmanov , X. Hu , A. Kasumov , F. Nori , Phys. Rev. B 2007, 75, 165417.

[166] P. Ying , J. Zhang , Z. Zhong , Microporous Mesoporous Mater. 2021, 312, 110765.

[167] D. J. Cerasale , D. C. Ward , T. L. Easun , Nat. Rev. Chem. 2022, 6, 9. 37117616 10.1038/s41570-021-00336-8

[168] S. Krause , J. V. Milić , Commun. Chem. 2023, 6, 151. 37452112 10.1038/s42004-023-00945-y PMC10349092

[169] J. D. Evans , V. Bon , I. Senkovska , H.-C. Lee , S. Kaskel , Nat. Commun. 2020, 11, 2690. 32483346 10.1038/s41467-020-16527-8 PMC7264271

[170] V. Van Speybroeck , S. Vandenhaute , A. E. Hoffman , S. M. Rogge , Trends Chem. 2021, 3, 605.

[171] A. Gonzalez‐Nelson , F.-X. Coudert , M. A. van der Veen , Nanomaterials 2019, 9, 330. 30832298 10.3390/nano9030330 PMC6474009

[172] J. Gonzalez , R. Nandini Devi , D. P. Tunstall , P. A. Cox , P. A. Wright , Microporous Mesoporous Mater. 2005, 84, 97.

[173] N. B. Shustova , T.-C. Ong , A. F. Cozzolino , V. K. Michaelis , R. G. Griffin , M. Dincă , J. Am. Chem. Soc. 2012, 134, 15061. 22889020 10.1021/ja306042w PMC3448963

[174] A. E. Khudozhitkov , D. I. Kolokolov , A. G. Stepanov , V. A. Bolotov , D. N. Dybtsev , J. Phys. Chem. C 2015, 119, 28038.

[175] D. I. Kolokolov , A. G. Stepanov , V. Guillerm , C. Serre , B. Frick , H. Jobic , J. Phys. Chem. C 2012, 116, 12131.

[176] A. E. Khudozhitkov , H. Jobic , D. I. Kolokolov , D. Freude , J. Haase , A. G. Stepanov , J. Phys. Chem. C 2017, 121, 11593.

[177] A. E. Khudozhitkov , H. Jobic , D. Freude , J. Haase , D. I. Kolokolov , A. G. Stepanov , J. Phys. Chem. C 2016, 120, 21704.

[178] D. I. Kolokolov , A. G. Stepanov , H. Jobic , J. Phys. Chem. C 2014, 118, 15978.

[179] D. Kolokolov , H. Jobic , A. Stepanov , V. Guillerm , T. Devic , C. Serre , G. Férey , Angew. Chem., Int. Ed. 2010, 49, 4791. 10.1002/anie.201001238 20512831

[180] S. L. Gould , D. Tranchemontagne , O. M. Yaghi , M. A. Garcia‐Garibay , J. Am. Chem. Soc. 2008, 130, 3246. 18288839 10.1021/ja077122c

[181] B. Cheng , T. Schumann , Y. Wang , X. Zhang , D. Barbalas , S. Stemmer , N. Armitage , Nano Lett. 2020, 20, 5991. 32633978 10.1021/acs.nanolett.0c01983

[182] M. Basini , M. Pancaldi , B. Wehinger , M. Udina , T. Tadano , M. Hoffmann , A. Balatsky , S. Bonetti , Nature, 2024, 628, 534. 38600387 10.1038/s41586-024-07175-9 PMC11023939

[183] F. G. G. Hernandez , A. Baydin , S. Chaudhary , F. Tay , I. Katayama , J. Takeda , H. Nojiri , A. K. Okazaki , P. H. O. Rappl , E. Abramof , M. Rodriguez‐Vega , G. A. Fiete , J. Kono , Sci. Adv. 2023, 9, eadj407. 10.1126/sciadv.adj4074 PMC10848715 38100589

[184] D. M. Juraschek , P. Narang , N. A. Spaldin , Rev. Res. 2020, 2, 043035.

[185] A. Baydin , F. G. G. Hernandez , M. Rodriguez‐Vega , A. K. Okazaki , F. Tay , G. T. Noe , I. Katayama , J. Takeda , H. Nojiri , P. H. O. Rappl , E. Abramof , G. A. Fiete , J. Kono , Phys. Rev. Lett. 2022, 128, 075901. 35244438 10.1103/PhysRevLett.128.075901

[186] C. P. Romao , R. Catena , N. A. Spaldin , M. Matas , Phys. Rev. Research 2023, 5, 043262.

[187] C. W. Misner , K. S. Thorne , J. A. Wheeler , Gravitation, Macmillan 1973.

[188] L. Ryder , Gen. Relativ. Gravitation 2008, 40, 1111.

[189] F. W. Hehl , W.-T. Ni , Phys. Rev. D 1990, 42, 2045. 10.1103/physrevd.42.2045 10013053

[190] R. M. Geilhufe , Phys. Rev. Res. 2022, 4, L012004.

[191] S. A. Werner , J. L. Staudenmann , R. Colella , Phys. Rev. Lett. 1979, 42, 1103.

[192] A. Danner , B. Demirel , W. Kersten , H. Lemmel , R. Wagner , S. Sponar , Y. Hasegawa , npj Quantum Inf. 2020, 6, 23.

[193] M. Xue , S. Ma , Z. Jin , R. M. Schaffino , G.-S. Zhu , E. B. Lobkovsky , S.-L. Qiu , B. Chen , Inorg. Chem. 2008, 47, 6825. 18582032 10.1021/ic800854y

[194] J. Hafizovic , M. Bjørgen , U. Olsbye , P. D. C. Dietzel , S. Bordiga , C. Prestipino , C. Lamberti , K. P. Lillerud , J. Am. Chem. Soc. 2007, 129, 3612. 17341071 10.1021/ja0675447

